# Fast Interfacial Hole Consumption Suppresses Space–Charge Layer Trap Filling in BiVO_4_ Photoanodes

**DOI:** 10.1021/jacs.6c05776

**Published:** 2026-06-19

**Authors:** Longren Li, Tong Wang, Beier Hu, Louise I. Oldham, Qiyun Chen, Keming Li, Chuxi Yang, Paransa Alimard, Zhu Meng, Haoqing Ning, James R. Durrant, Andreas Kafizas, Artem A. Bakulin

**Affiliations:** † Department of Chemistry, 4615 Imperial College London, Molecular Science Research Hub, 82 Wood Lane, White City Campus, London W12 0BZ, U.K.; ‡ Chemistry Research Laboratory, 6396 University of Oxford, Oxford OX1 3TA, U.K.; § London Centre for Nanotechnology, South Kensington Campus, 4615 Imperial College London, London SW7 2AZ, U.K.

## Abstract

Photoelectrochemical (PEC) oxidation of biomass-derived organics (e.g., glycerol) can outperform water oxidation while coproducing value-added chemicals. However, the kinetic basis of this enhanced performance, such as hole consumption dynamics and space–charge–layer (SCL) trap filling under PEC operating conditions, remains poorly understood. Using BiVO_4_ as a model photoanode, we combine operando optical and photocurrent spectroscopies, including trap-selective pump-push photocurrent (PPPC) mapping, to track bulk and interfacial charge-carrier dynamics over the femtosecond-to-second (fs–s) time scale. Overall, we show that glycerol oxidation accelerates interfacial hole consumption, lowering the surface-hole density required to sustain a given photocurrent, thereby suppressing SCL trap filling and trap-mediated recombination. Glycerol increases the per-hole turnover frequency 32-fold (∼3.5 to ∼113.2 s^–1^) and the photocurrent density at 1.23 V_RHE_ from ∼0.5 to ∼1.3 mA cm^–2^, while formic acid and dihydroxyacetone are the dominant quantified liquid products. Spatially resolved PPPC mapping (over ∼20 mm^2^) shows that glycerol also suppresses localized trap-filled hot spots. Glycerol leaves the dominant early time bulk carrier dynamics largely unchanged while suppressing the microsecond buildup of trapped electrons in the SCL. These results highlight microsecond time-scale kinetic competition between interfacial hole consumption and SCL trap filling as a key design principle for PEC oxidation of renewable organics.

## Graphical abstract

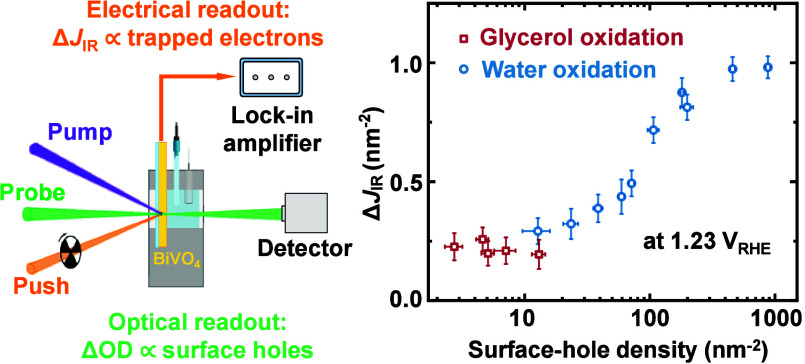

## Introduction

Photoelectrochemical (PEC) water splitting offers a sustainable route to solar-to-hydrogen conversion, but its efficiency is often constrained by the sluggish oxygen evolution reaction (OER) at the photoanode.^1–3^ Since OER requires the accumulation and coupled transfer of four oxidative equivalents, it typically proceeds on the millisecond-to-second (ms-s) time scale, whereas photogenerated carriers in metal-oxide photoanodes can localize and recombine on much shorter picosecond-to-nanosecond (ps-ns) time scales.^4^ Under OER conditions, sustaining an anodic photocurrent often requires significant hole accumulation at the semiconductor/electrolyte interface; however, the persistent interfacial holes can also promote back electron–hole recombination (BER), thereby limiting the achievable PEC efficiency.^5–7^ This kinetic mismatch is particularly severe in earth-abundant metal oxides, including TiO_2_, α-Fe_2_O_3_, WO_3_, and BiVO_4_, which are attractive candidates for scalable PEC devices owing to their low cost and durability under oxidative aqueous conditions.^8–11^ Bismuth vanadate (BiVO_4_) exemplifies this mismatch as a benchmark visible-light photoanode where surface-hole accumulation has been linked to pronounced surface recombination.^12^ Accordingly, BiVO_4_ has emerged as a model photoanode for evaluating strategies that modulate interfacial hole consumption and improve photocurrent, ranging from catalyst/passivation overlayers (e.g., NiFeOOH) to fast hole scavengers (e.g., glycerol).^13–16^ Despite extensive electrochemical and spectroscopic studies, strategies that accelerate interfacial oxidation can also change subsurface charge-loss kinetics, complicating mechanistic assignment and motivating a unified kinetic framework that explicitly links interfacial oxidation to near-surface carrier-loss pathways.^17–21^ More broadly, trap states are increasingly recognized as key factors governing BiVO_4_ PEC performance, particularly at low bias, where charge transport can become trap-mediated and strongly influenced by small-polaron hopping.^22^ Among these loss pathways, space-charge-layer (SCL) trap filling and the associated trap-mediated losses have rarely been quantified directly under operando PEC conditions, even though the resulting trapped-electron population can mediate interfacial recombination and limit charge collection.^23,24^

In addition to recombination through interfacial surface states, spectroscopic studies have implicated oxygen-vacancy-associated sub-bandgap states in BiVO_4_ as near-surface electron traps that can become occupied within the SCL region and mediate trap-assisted recombination. As the conduction-band edge in BiVO_4_ is primarily of vanadium 3d character, oxygen-vacancy formation can localize electrons on neighboring vanadium sites, reducing them from V^5+^ to V^4+^.^25,26^ Here, we denote the corresponding vacancy-associated trap states as V_OV_^5+^ (unoccupied) and V_OV_^4+^ (electron-occupied). By modulating the occupancy of this V_OV_^4+^/V_OV_^5+^ distribution, Selim et al. showed that vacancy ionization/electron detrapping is thermally activated (*E*_a_ ≈ 200 meV) and can govern the kinetics of electron extraction.^23^ More recently, trap-selective pump-push photocurrent (PPPC) spectroscopy, in which an infrared “push” laser reactivates trapped electrons, has provided an operando electrical readout of trap filling in the SCL. Applied to BiVO_4_, PPPC revealed that the SCL trapped-electron population associated with this V_OV_^4+^/V_OV_^5+^ distribution remains recombination-active even under strong interfacial band bending, with trap-filling dynamics spanning from <10 ns to ∼100 μs.^24^ However, how the SCL trapped-electron buildup responds to changes in interfacial oxidation chemistry (e.g., from water oxidation to faster organic oxidation) remains poorly understood under operando conditions. Complementary temperature-dependent transient measurements further suggest that BER is associated with the same V_OV_^4+^/V_OV_^5+^ distribution.^12^ The largely potential-independent activation barrier for BER (∼60–100 meV) is substantially smaller than the ∼200 meV detrapping barrier associated with electron extraction,^23^ consistent with recombination competing favorably with detrapping into the extraction pathway. Here, BER denotes recombination between accumulated interfacial holes and electrons supplied from the semiconductor/back-contact side, with SCL trapped electrons treated as the relevant electron population. Taken together, these studies point toward a kinetic competition within the near-surface SCL of BiVO_4_: trapped electrons associated with the V_OV_^4+^/V_OV_^5+^ distribution can either be detrapped and extracted or retained in the SCL, where they mediate recombination with surface holes. Interfacial hole-consumption kinetics modulate the density and persistence of accumulated surface holes; changing the interfacial oxidation chemistry therefore allows us to test how this hole population shapes SCL trapped-electron buildup and the associated trap-mediated recombination under operando PEC conditions.

Within this framework, PEC oxidation of biomass-derived organics (e.g., glycerol, glucose, 5-hydroxymethylfurfural, and furfural) provides a practical way to vary interfacial hole consumption kinetics while enabling value-added anodic chemistry.^13,27–32^ Among these substrates, glycerol is especially attractive because it is an abundant, renewable byproduct of biodiesel production and can be upgraded to high-value C3 products such as dihydroxyacetone (DHA).^27,33–35^ Consistent with this promise, glycerol addition has been widely reported to increase photocurrent densities and cathodically shift onset potentials on BiVO_4_ and other oxide photoanodes. Recent studies further show that product selectivity can be tuned through the use of cocatalysts and interfacial engineering.^13,35–40^ This PEC performance improvement is commonly attributed to faster interfacial hole consumption, although the underlying carrier kinetics remain poorly understood.^19,41,42^ Conventional steady-state and impedance-based analyses do not directly resolve the temporal relationship among surface-hole accumulation, SCL trap filling, and photocurrent generation during glycerol PEC oxidation. Resolving this relationship requires operando, time-resolved measurements that quantify surface-hole accumulation, probe trapped-electron buildup, and correlate both with the photocurrent under PEC operating conditions.

Here, we establish an operando, time-resolved kinetic framework linking interfacial hole consumption to SCL trap filling in the BiVO_4_ photoanodes. By combining transient absorption (TA), photoinduced absorption (PIA), transient photocurrent (TPC), and trap-selective PPPC, we track early time bulk carrier dynamics, surface-hole accumulation, interfacial charge-transfer flux, and SCL trapped-electron buildup across the femtosecond-to-second (fs–s) time window. We show that glycerol oxidation accelerates interfacial hole consumption, thereby reducing the surface-hole density required to sustain a given photocurrent and suppressing microsecond SCL trap filling while leaving the dominant early time bulk carrier dynamics largely unchanged.

## Results and Discussion

### Characterization of the BiVO_4_ Photoanode

We first prepared BiVO_4_ photoanodes on fluorine-doped tin oxide (FTO) substrates by aerosol-assisted chemical vapor deposition (AA-CVD).^43–45^ The resulting films show XRD patterns consistent with phase-pure monoclinic scheelite BiVO_4_,^46^ with no detectable impurity peaks (Figure S1). The optical response is consistent with this phase assignment: absorbance is negligible above ∼500 nm, rises sharply near ∼490 nm, and exhibits a characteristic feature near ∼440 nm (Figure S2). The corresponding Tauc analysis yields an optical band gap of *E*_g_ ≈ 2.5 eV (Figure S3),^25^ consistent with the expected visible-light absorption window of BiVO_4_. Scanning electron microscopy (SEM) further confirms the compact morphology and film continuity. Top-view SEM images show a compact, densely packed granular surface with no obvious pinholes or large cracks (Figure S4), while cross-sectional SEM images show a continuous BiVO_4_ layer conformally covering the FTO substrate, with an average thickness of ∼550 nm (Figure S5). Together, these basic structural and optical characteristics establish a phase-pure, continuous BiVO_4_ photoanode and provide a consistent baseline for comparing operando interfacial hole-consumption and SCL trap-filling dynamics across electrolyte conditions.

### Glycerol-Enhanced PEC Performance of BiVO_4_ Photoanodes

Under backside (FTO-side) illumination with a 365 nm LED calibrated to a one-sun-equivalent incident photon flux (see Note S2.1), adding 1.0 M glycerol to 0.5 M Na_2_SO_4_ (pH 6) markedly enhances the PEC performance of BiVO_4_ photoanodes. Glycerol increases the photocurrent across the measured potential range and shifts the apparent onset to lower potentials (Figure 1a). At 1.23 V_RHE_, the photocurrent density increases from ∼0.5 to ∼1.3 mA cm^–2^ (2.6-fold), while the onset potential shifts cathodically from ∼0.7 to ∼0.5 V_RHE_, showing that higher photocurrent densities can be reached at lower applied potentials in the presence of glycerol. Such a cathodic onset shift is consistent with reduced apparent interfacial kinetic limitation and/or suppressed interfacial losses under glycerol oxidation relative to water oxidation. Dark *J*–*V* curves remain near-zero and featureless within 0.3–1.5 V_RHE_ (<0.001 mA cm^–2^ for both electrolyte conditions), and appreciable dark anodic currents appear only above ∼2.0 V_RHE_ (Figure S6), indicating that glycerol does not generate a substantial electrocatalytic anodic current. The glycerol-induced photocurrent enhancement and cathodic onset potential shift are also observed under AM 1.5G illumination (Figure S7). The glycerol concentration-dependent measurements show a progressive increase in the photocurrent and cathodic onset potential shift from 0 to 1.0 M glycerol, with only modest additional improvement at higher concentrations (Figure S8). Continuous chronoamperometry (CA) at 1.23 V_RHE_ further shows that the higher photocurrent in 1.0 M glycerol is maintained over the 2 h test period (Figure S9).

**Figure 1:**
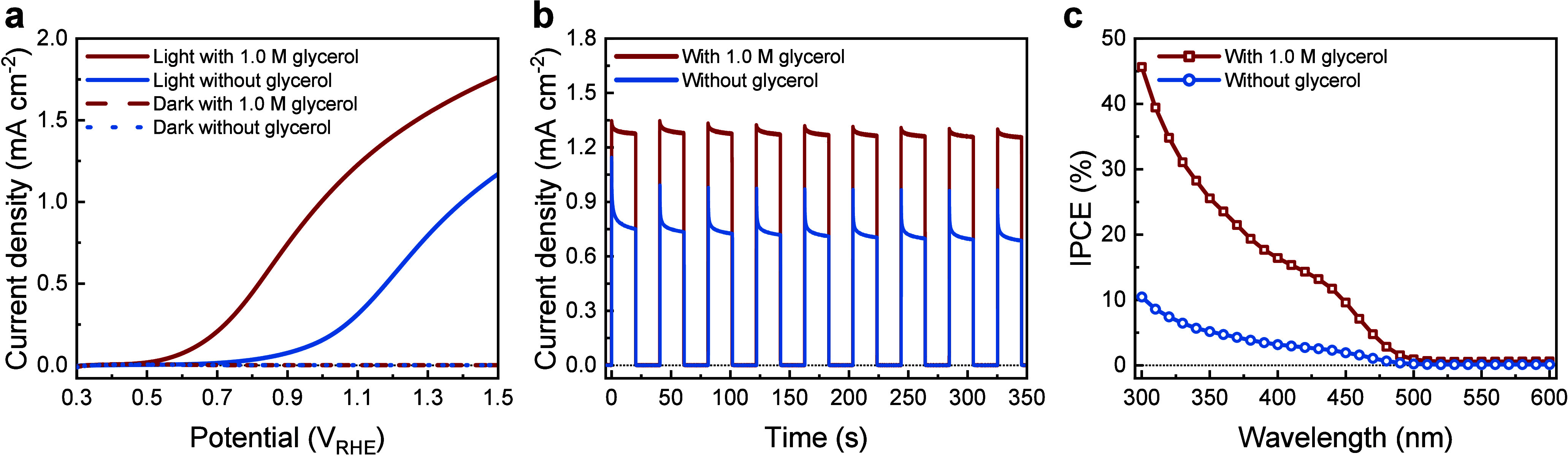
Glycerol-enhanced PEC performance of BiVO_4_ photoanodes. (a) Current density–potential (*J*–*V*) curves of BiVO_4_ measured under backside illumination (through the FTO side) using a 365 nm LED calibrated to a one-sun-equivalent incident photon flux (see Note S2.1), in 0.5 M Na_2_SO_4_ (pH 6) with and without 1.0 M glycerol; corresponding dark scans are shown for comparison. (b) Chopped-light CA recorded at 1.23 V_RHE_ under the same electrolyte and illumination conditions. (c) IPCE spectra measured at 1.23 V_RHE_ in the same electrolytes under backside monochromatic illumination.

Chopped-light CA at 1.23 V_RHE_ further highlights distinct transient signatures (Figure 1b). With 1.0 M glycerol, the photocurrent rises rapidly to a higher plateau and shows only a minor decay during each light-on period. Without glycerol, the photocurrent reaches a lower plateau and decays substantially during illumination, consistent with stronger interfacial charge accumulation and associated recombination losses. Chopped-light *J*–*V* measurements (Figure S10) provide an additional kinetic signature for recombination losses. Without glycerol, a clear light-off cathodic overshoot transient persists up to ∼1.3 V_RHE_, whereas in the presence of 1.0 M glycerol this negative spike is attenuated beyond ∼ 0.9 V_RHE_. These cathodic overshoots are commonly assigned to the BER-driven discharge of accumulated interfacial charge.^7^ The earlier attenuation of the cathodic overshoot upon glycerol addition is consistent with faster interfacial hole consumption, reduced surface-hole accumulation, and suppression of the BER pathway at a substantially lower potential. Figure 2 further supports this interpretation.

**Figure 2:**
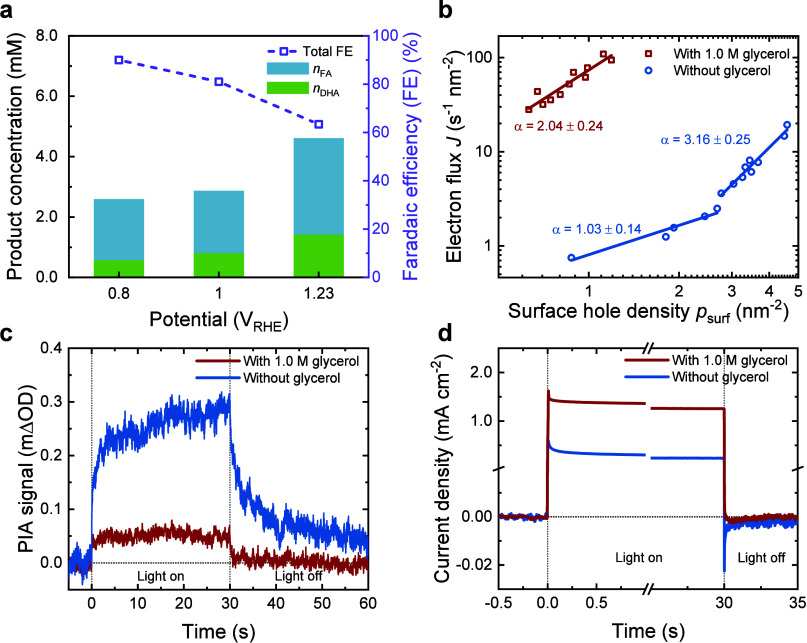
Product distribution and interfacial charge-transfer kinetics during glycerol PEC oxidation. (a) Concentrations of FA and DHA quantified by HPLC after a 2 h PEC operation at the indicated potentials in 10 mL of 0.5 M Na_2_SO_4_ (pH 6) containing 1.0 M glycerol under backside one-sun-equivalent 365 nm LED illumination. The overlaid “total FE” corresponds to the combined FE for the quantified liquid products, FE_total_ = FE_FA_ + FE_DHA_. (b) Rate-law analysis at 1.23 V_RHE_ in 0.5 M Na_2_SO_4_ (pH 6) with and without 1.0 M glycerol: electron flux (*J*) extracted from the quasi-steady-state photocurrent in TPC measurements plotted against the surface-accumulated hole density (*p*_surf_) extracted from the calibrated PIA signal amplitude at 550 nm. Solid lines are power-law fits, *J* = *kp*_surf_^α^, with fitted apparent reaction orders α. (c) 550 nm PIA response to a square 365 nm light pulse (one-sun-equivalent) at 1.23 V_RHE_ in 0.5 M Na_2_SO_4_ (pH 6) with and without 1.0 M glycerol. (d) Corresponding TPC response to the same 365 nm light pulse measured simultaneously under the same conditions as in (c).

Incident photon-to-electron conversion efficiency (IPCE) spectra indicate that glycerol improves charge-carrier utilization within the BiVO_4_ photoactive window, without introducing a detectable sub-bandgap photoresponse (Figure 1c). In both electrolyte conditions, the IPCE is sharply bandgap-limited, falling to ∼0% at wavelengths longer than ∼ 500 nm (photon energies below ∼2.5 eV), consistent with the supra-bandgap excitation of BiVO_4_ and the absence of any detectable sub-bandgap photoresponse (Figure S3). At 1.23 V_RHE_, glycerol increases the IPCE across the full photoactive window (∼300–490 nm); for example, the IPCE at 400 nm rises from ∼3 to ∼16%. Under backside illumination, a higher IPCE at shorter wavelengths is expected as more strongly absorbed photons generate carriers closer to the FTO/BiVO_4_ interface with a shortened electron transport distance.

### Product Distribution and Charge Utilization

High-performance liquid chromatography (HPLC) analysis of the postreaction electrolyte quantifies the major identified liquid products formed during glycerol PEC oxidation. After 2 h of backside illumination in 0.5 M Na_2_SO_4_ (pH 6) containing 1.0 M glycerol, formic acid (FA) and DHA are the dominant quantified products (Figure 2a). Their concentrations increase with applied potential from 0.8 to 1.23 V_RHE_, consistent with the higher total oxidation turnover at higher potential. Across this potential window, FA remains the major quantified product (∼69–78% of FA + DHA), with DHA contributing ∼22–31%. The codetection of DHA and FA is consistent with previous reports of glycerol oxidation on BiVO_4_-based photoanodes. DHA, as a C3 product, is generally associated with partial oxidation of glycerol without C–C bond cleavage, including oxidation of the secondary alcohol to a ketone, whereas FA, as a C1 product, reflects C–C bond cleavage and is commonly associated with deeper oxidation of glycerol-derived intermediates.^33,34,36,39^ The total Faradaic efficiency (FE) for FA + DHA formation is highest at moderate potential (∼90% at 0.8 V_RHE_) and decreases at higher potential (∼63% at 1.23 V_RHE_) (Figure 2a; see Note S2.2 for FE calculations and analytical details). This potential dependence is consistent with an increasing contribution from unquantified parallel pathways at higher potential (e.g., further oxidation to undetected products and/or competing OER), although a full carbon and gas-phase balance is not specified in the present data set.

### Rate-Law Analysis of Surface-Hole Reaction Kinetics

To connect product formation to interfacial reaction kinetics, we combined operando PIA and TPC measurements over a range of illumination intensities and correlated the quasi-steady-state surface-hole density (*p*_surf_) with the extracted electron flux (*J*) (see Note S2.3). Following established analyses for BiVO_4_,^19^ the data are described by

$$J=k{p_{\mathrm{surf}}}^{\alpha }$$ 1

where α is the apparent reaction order and *k* is the apparent rate constant. Without glycerol (i.e., water oxidation), *J* shows two kinetic regimes (Figure 2b): at low *p*_surf_, *J* scales approximately linearly with *p*_surf_ (α ≈ 1), whereas at higher *p*_surf_, the dependence becomes strongly supralinear (α ≈ 3), consistent with the onset of multihole chemistry required for a faster OER turnover.^19^ With 1.0 M glycerol, the *J* is described by a single power-law over the measured range with an apparent second-order dependence (α ≈ 2). Notably, at a given *J*, glycerol enables substantially lower *p*_surf_ than water oxidation, indicating a kinetically distinct hole-consumption pathway that does not require sustaining a large steady-state surface-hole population to achieve high photocurrent.

The 550 nm PIA signal, assigned to accumulated surface holes,^6,19,20^ and the TPC were recorded simultaneously in response to a 30 s square 365 nm excitation pulse at 1.23 V_RHE_ in 0.5 M Na_2_SO_4_ (pH 6), in the absence and presence of 1.0 M glycerol (Figure 2c,d). In glycerol-free electrolyte, the PIA signal exhibits a rapid initial rise followed by a continued slow increase throughout the light-on period, reaching its maximum ∼0.29 mΔOD at 30 s. Using the PIA calibration, this corresponds to a substantially larger quasi-steady-state surface-hole density (≈4.2 holes nm^–2^). After the excitation light is switched off, the PIA signal decays slowly and remains above the baseline even after 60 s, indicating sluggish interfacial hole consumption and a long-lived population of accumulated surface holes under water-oxidation conditions. In the presence of 1.0 M glycerol, both the amplitude and persistence of the PIA signal are markedly reduced. The PIA signal rises quickly to a much smaller steady value (∼0.05 mΔOD at 30 s; ≈0.7 holes nm^–2^) and then returns to baseline rapidly after light-off. Thus, glycerol sustains a higher photocurrent density (Figure 2d) while requiring a much lower quasi-steady-state surface-hole density, indicating that the higher photocurrent arises from faster interfacial hole consumption (shorter surface-hole residence time) rather than from building up a larger surface-hole population. Quantitatively, the per-hole turnover frequency (TOF) at 1.23 V_RHE_ under one-sun-equivalent illumination increases from ∼3.5 s^–1^ without glycerol to ∼113.2 s^–1^ with 1.0 M glycerol, indicating substantially faster apparent consumption of accumulated surface holes in the presence of glycerol. These values were extracted at 30 s from the quasi-steady-state PIA and photocurrent data in Figure 2c,d (see Note S2.4). Consistent with the PIA results, the TPC traces also show a clear effect of glycerol on interfacial charge dynamics. Under illumination, glycerol increases the quasi-steady-state photocurrent at 30 s due to efficient interfacial hole consumption. Upon light-off, the glycerol-free electrolyte shows a pronounced negative current peak, commonly attributed to recombination-driven discharge of accumulated interfacial charge via BER.^7^ This light-off cathodic overshoot is strongly attenuated in the presence of glycerol, with no discernible negative current peak within our detection limit, consistent with reduced surface-hole accumulation and recombination losses.

Taken together, these results indicate that glycerol oxidation provides a kinetically facile pathway for interfacial hole consumption on BiVO_4_ (relative to that of water oxidation). This faster interfacial reaction enables a given photocurrent to be sustained at a substantially lower quasi-steady-state surface-hole density and, at 1.23 V_RHE_, translates into a markedly higher measured photocurrent together with strong suppression of the light-off cathodic overshoot commonly attributed to BER. We next use trap-selective PPPC measurements (Figures 3 and 4) to examine how faster interfacial hole consumption suppresses SCL trapped-electron buildup under operando conditions.

**Figure 3:**
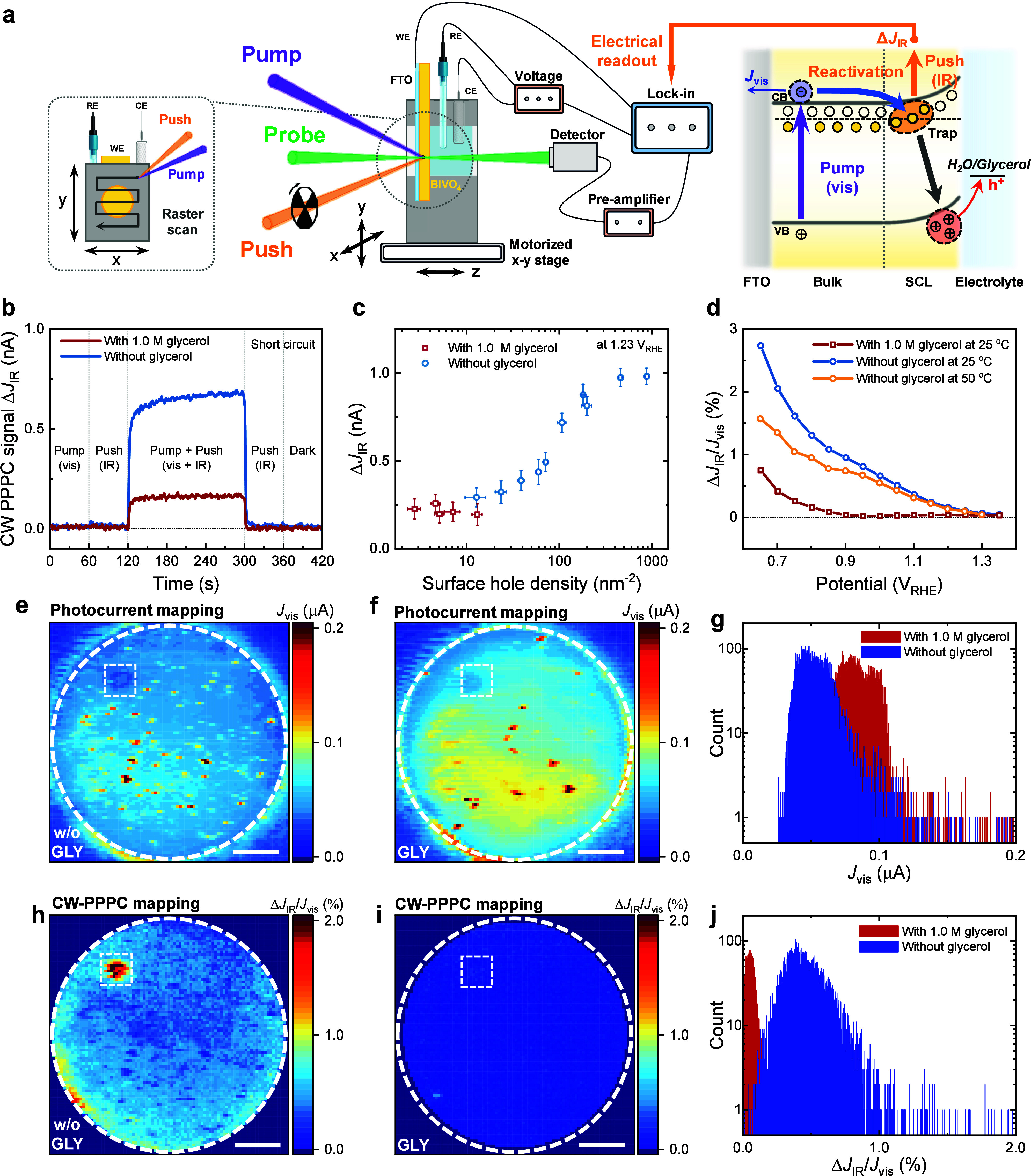
CW-PPPC measurements and spatial mapping in BiVO_4_ photoanodes. (a) Schematic of the operando CW-PPPC setup and measurement principle. (b) Representative time traces of Δ*J*_IR_ recorded under short-circuit conditions in 0.5 M Na_2_SO_4_ (pH 6) with and without 1.0 M glycerol. The pump intensity is 0.015 W cm^–2^, and the push intensity is 0.85 W cm^–2^. (c) Correlation between the surface-hole density (*p*_surf_) quantified from the concurrently measured 550 nm PIA signal and the corresponding Δ*J*_IR_ measured at 1.23 V_RHE_ in 0.5 M Na_2_SO_4_ (pH 6) with and without 1.0 M glycerol. The pump intensity was varied (see Experimental Details S1.7), and the push intensity was fixed at 0.85 W cm^–2^. (d) Potential dependence of Δ*J*_IR_/*J*_vis_, where *J*_vis_ is the pump-only photocurrent, measured in 0.5 M Na_2_SO_4_ (pH 6) without glycerol (25 and 50 °C) and with 1.0 M glycerol (25 °C). (e, f) Spatial maps of the *J*_vis_ measured at 1.23 V_RHE_ in 0.5 M Na_2_SO_4_ (pH 6) without and with 1.0 M glycerol, respectively. (g) Corresponding pixel-value histograms of *J*_vis_. (h, i) Spatial maps of Δ*J*_IR_/*J*_vis_ for the same sample area measured at 1.23 V_RHE_ in 0.5 M Na_2_SO_4_ (pH 6) without and with 1.0 M glycerol, respectively. (j) Corresponding pixel-value histograms of Δ*J*_IR_/*J*_vis_. For mapping, the effective optical spot diameter is ∼120 μm, much larger than the submicrometer BiVO_4_ grains; therefore, each mapped pixel shows an averaged operando response from many grains. The pump intensity is 0.14 W cm^–2^, and the push intensity is 0.85 W cm^–2^. White dashed circles delineate the illuminated/active area; the white dashed box marks the same inhomogeneous area for comparison across maps; scale bar, 1 mm. All measurements use backside (through the FTO side) illumination.

**Figure 4:**
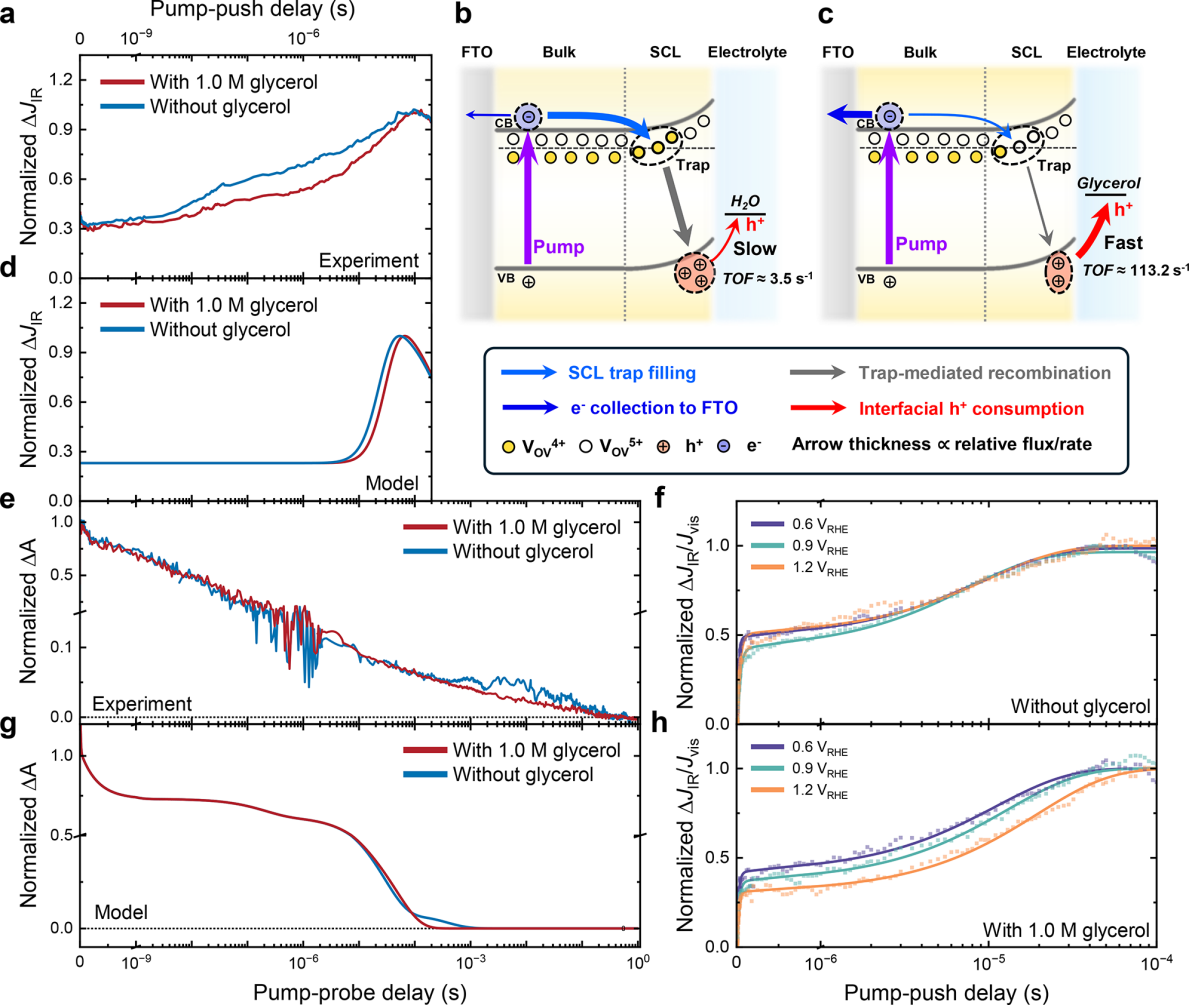
Time-resolved charge-carrier dynamics and mechanistic interpretation of glycerol-induced suppression of SCL trap filling in BiVO_4_. (a) TR-PPPC transients normalized to the maximum signal reached before decay, measured at 1.2 V_RHE_ in 0.5 M Na_2_SO_4_ (pH 6) with and without 1.0 M glycerol as a function of pump-push delay. (b, c) Schematic illustrations comparing the BiVO_4_ photoanode response under water-oxidation and glycerol-oxidation conditions. (d) Modeled evolution of the trapped-electron population in the SCL. (e) Normalized transient absorption kinetics extracted at 550 nm, tracking the hole population over fs–s time window measured at 1.2 V_RHE_ in 0.5 M Na_2_SO_4_ (pH 6) with and without 1.0 M glycerol. Full TA spectra are shown in Figures S18 and S19. (f) Potential-dependent Δ*J*_IR_/*J*_vis_ transients, normalized to their maximum signal reached before decay, recorded without glycerol at the indicated applied potentials. (g) Modeled evolution of the hole population. (h) Potential-dependent Δ*J*_IR_/*J*_vis_ transients, normalized to their maximum signal reached before decay, recorded with 1.0 M glycerol at the indicated applied potentials. All illumination conditions are given in the SI Experimental Details.

### Surface-Hole-Controlled SCL Trap Filling Revealed by PPPC Spectroscopy

Figure 3a illustrates the continuous-wave PPPC spectroscopy (CW-PPPC) operating principle used here to compare the trapped-electron response in BiVO_4_ under water- and glycerol-oxidation conditions. A 405 nm “pump” generates charge carriers, giving rise to the pump-only photocurrent, *J*_vis_. A modulated 980 nm sub-bandgap “push” photoionizes a subset of trapped electrons into mobile states, producing an additional extracted photocurrent increment, Δ*J*_IR_. This increment is defined as the CW-PPPC response and is detected at the push-modulation frequency using a lock-in amplifier. Since Δ*J*_IR_ requires both trapped-electron reactivation and subsequent carrier extraction; it serves as an operando readout of the quasi-steady-state population of trapped electrons that eventually contribute to recombination losses. In BiVO_4_, this Δ*J*_IR_ has been established as an indicator of the average concentration of electrons trapped in vacancy states within the SCL.^24^ Simultaneously, the 550 nm “probe” monitors the PIA signal assigned to accumulated surface holes, enabling direct comparison of surface-hole densities and Δ*J*_IR_ across different electrolyte conditions.

Figure 3b shows representative pump/push on–off traces of Δ*J*_IR_ measured with and without 1.0 M glycerol under lower-intensity excitation (∼one-sun-equivalent conditions) to verify the origin of the PPPC signal. When either the “pump” or the “push” is applied individually, the lock-in amplifier detected Δ*J*_IR_ remains at the baseline, whereas a finite response is observed only when both beams are present. This confirms that Δ*J*_IR_ arises from push-induced reactivation of pump-generated trapped electrons, rather than from direct carrier generation by the “push” alone. Control measurements of the push-intensity dependence further show that Δ*J*_IR_ scales approximately linearly with push intensity in both electrolyte conditions over the investigated range (Figure S11), confirming that the Δ*J*_IR_ was acquired in the linear, unsaturated regime. After establishing the signal origin under lower-intensity excitation, we performed the CW-PPPC correlation, potential-dependent, and spatial-mapping measurements, as shown in Figure 3c–j under higher excitation density to obtain an appreciable trapped-electron response at high applied potentials. These higher-intensity CW-PPPC measurements are therefore used to probe relative changes in the SCL trapped-electron buildup, rather than to quantify absolute trap occupancy under one-sun-equivalent operating conditions.

To examine how the SCL trap filling relates to surface-hole accumulation, we varied the pump intensity while keeping the push intensity constant and simultaneously measured Δ*J*_IR_ and the quasi-steady-state surface-hole density (*p*_surf_) from the calibrated 550 nm PIA amplitude (Figure 3c). In glycerol-free electrolyte, Δ*J*_IR_ changes only weakly at low *p*_surf_ but increases more strongly at higher *p*_surf_ (Figure 3c). As the pump intensity increases during water oxidation, the higher hole flux is sustained by a progressively larger quasi-steady-state surface-hole population. At low *p*_surf_, this accumulated hole population is insufficient to appreciably increase the SCL trapped-electron buildup; at higher *p*_surf_, however, it favors SCL trap filling and gives rise to the larger Δ*J*_IR_ response. Given the linear push-intensity dependence, the plateau in Δ*J*_IR_ at the highest *p*_surf_ values is consistent with the PPPC-detectable SCL trapped-electron population approaching a limiting value under these measurement conditions. In contrast, 1.0 M glycerol maintains the BiVO_4_ in a low-*p*_surf_/low-Δ*J*_IR_ regime over the measured intensity range, consistent with faster interfacial hole consumption limiting surface-hole accumulation and suppressing SCL trap filling.

To further examine the kinetic factors governing SCL trap filling, we performed potential- and temperature-dependent CW-PPPC measurements (Figure 3d). Since potential and temperature can also change the number of pump-generated electrons available for trapping, we normalized Δ*J*_IR_ by the corresponding *J*_vis_ measured under identical conditions. The resulting ratio, Δ*J*_IR_/*J*_vis_ (%), is therefore used here as a practical comparative indicator of the relative trapped-electron population in the SCL. Figure 3d summarizes the Δ*J*_IR_/*J*_vis_ measured from 0.65 to 1.35 V_RHE_ with and without 1.0 M glycerol. At 25 °C, Δ*J*_IR_/*J*_vis_ decreases with increasing anodic potential under both electrolyte conditions, indicating that the SCL trapped-electron buildup becomes progressively less significant at more positive potentials. Glycerol suppresses Δ*J*_IR_/*J*_vis_ across the full potential window and drives it close to zero already at ∼0.9 V_RHE_, whereas in glycerol-free electrolyte, Δ*J*_IR_/*J*_vis_ approaches near-zero only at ∼1.3 V_RHE_. Thus, glycerol shifts BiVO_4_ into the low-SCL trap-filling regime at a substantially lower potential. Notably, this potential shift closely matches the disappearance of the light-off cathodic overshoot in chopped-light LSV (Figure S10): the overshoot is strongly attenuated above ∼0.9 V_RHE_ with glycerol, whereas only above ∼1.3 V_RHE_ without glycerol. This correspondence between these two independent electrical observables suggests that the CW-PPPC response and the light-off cathodic overshoot are sensitive to a related interfacial loss process. From a performance perspective, the *J*–*V* response in Figure 1a shows that the cathodically shifted photocurrent onset with glycerol coincides with the potential range in which Δ*J*_IR_/*J*_vis_ becomes strongly suppressed. We then used temperature as an additional kinetic perturbation in glycerol-free electrolyte (Figure 3d). Heating the electrolyte from 25 to 50 °C lowers Δ*J*_IR_/*J*_vis_ across the measured potential window but not to the same extent as adding 1.0 M glycerol at 25 °C. This shows that SCL trap filling is sensitive to thermally activated processes.

BiVO_4_ films can exhibit mesoscale photoactivity heterogeneity arising from variations in defect density, charge-transport pathways, and local contact resistance.^47–49^ To examine how SCL trap filling correlates with the local photocurrent across this heterogeneity, we performed CW-PPPC mapping of both *J*_vis_ (Figure 3e,f) and Δ*J*_IR_/*J*_vis_ (Figure 3h,i), over the same ∼20 mm^2^ area with a step size of ∼50 μm, in the absence and presence of 1.0 M glycerol. Without glycerol, the *J*_vis_ map is clearly spatially nonuniform (Figure 3e), exhibiting a broad pixel-value distribution (Figure 3g) and a distinct mesoscale low-photocurrent feature (∼0.07 mm^2^) within the dashed-box region. The corresponding Δ*J*_IR_/*J*_vis_ map is even more inhomogeneous (Figure 3h): the same region shows elevated Δ*J*_IR_/*J*_vis_ relative to the surrounding area. This spatial colocalization provides direct functional evidence that lower local photocurrent is associated with a stronger SCL trapped-electron buildup. We therefore define the dashed-box region as a mesoscale trap-filled hot spot. Additional pump-intensity-dependent measurements show that Δ*J*_IR_ at this hot spot saturates at a higher value than in a nearby normal region (Figure S12a), indicating a larger accessible SCL trap-state capacity in the hot-spot region. Upon addition of 1.0 M glycerol, the *J*_vis_ map shifts to higher values and becomes markedly more uniform across the mapped area (Figure 3f,g). In parallel, the Δ*J*_IR_/*J*_vis_ map decreases to near-uniformly low values, including in the dashed-box region, where the hot spot is no longer resolved (Figure 3i,j)**.** Thus, although this region shows a larger accessible SCL trap-state capacity, glycerol suppresses trapped-electron buildup there, demonstrating that faster surface-hole consumption mitigates local trap-filling losses that would otherwise limit photocurrent output.

The raw Δ*J*_IR_ maps also show the glycerol-induced suppression (Figure S12b,c), confirming that the reduced Δ*J*_IR_/*J*_vis_ is not a normalization artifact caused by changes in *J*_vis_. Similar suppression is observed with other hole scavengers: both 10% methanol and 0.1 M Na_2_SO_3_ reduce Δ*J*_IR_ to a weak, nearly featureless background across the mapped area (Figure S13). Spatial heterogeneity in BiVO_4_ photoactivity has also been reported by high-resolution scanning electrochemical microscopy (SECM),^48,49^ which maps local interfacial reactivity but does not selectively probe the SCL trapped electrons. Our PPPC mapping provides a complementary operando functional readout, identifying mesoscale regions where stronger SCL trap filling coincides with lower photocurrent output. This operando mapping extends the single-point PPPC result by showing that glycerol suppresses SCL trap filling within mesoscale high-trap-capacity regions that would otherwise show stronger trapped-electron buildup and lower photocurrent output.

### Microsecond Time-Scale Buildup of the Trapped-Electron Population in the SCL

To identify the time scale over which the glycerol-induced kinetic differences emerge, we combined operando time-resolved PPPC (TR-PPPC) spectroscopy with fs–s transient absorption (TA) spectroscopy. TR-PPPC provides a time-resolved readout of the SCL trapped-electron population associated with recombination losses. At 1.2 V_RHE_, the normalized TR-PPPC transients exhibit a multiphasic rise under both electrolyte conditions (Figure 4a). This behavior is consistent with previous TR-PPPC studies of BiVO_4_, in which the response was described as comprising an early time (<1 μs) local trapping component and a slower microsecond component associated with transport-assisted buildup of SCL trapped electrons.^24^ When normalized at 1 μs to compare relative buildup kinetics, the traces overlap at early times and diverge only after ∼3 μs (Figure S14). This early time overlap indicates that glycerol does not measurably alter the initial local trapping. Instead, the glycerol-dependent response emerges in the later microsecond regime, where the buildup of trapped electrons in the SCL is delayed and the absolute Δ*J*_IR_ amplitude is reduced (Figure S15).

The 550 nm TA signal provides a complementary probe of the photogenerated-hole population (Note S2.19), with the early time response dominated by bulk holes and the longer-time response increasingly sensitive to persistent surface holes.^6^ For both electrolyte conditions, the TA kinetics are essentially indistinguishable up to the early microsecond regime (Figure 4e), indicating that glycerol does not measurably change the dominant early time bulk-hole dynamics. A clear difference emerges only at longer times (>∼500 μs): the long-lived TA tail observed in glycerol-free electrolyte is strongly attenuated in the presence of glycerol. This attenuation is consistent with faster consumption of accumulated surface holes, in agreement with the higher per-hole TOF under glycerol oxidation. Notably, the two measurements resolve the glycerol-dependent response in different temporal windows: TR-PPPC detects a glycerol-dependent difference in SCL trap filling after ∼3 μs, whereas the TA kinetics show a difference only at longer times (>∼500 μs). This temporal offset can be understood from the different carrier populations probed by the two techniques. TR-PPPC selectively resolves the SCL trapped electrons, whereas the TA signature of surface holes emerges only after the dominant bulk-hole signal has decayed. Thus, the later TA difference corresponds to the time window in which the surface-hole signal becomes detectable, rather than to the onset of glycerol-accelerated surface-hole consumption.

Figure 4b,c integrates the mechanistic picture established by the PIA/TPC, CW-PPPC, TR-PPPC, and TA measurements. Water oxidation requires a large and persistent surface-hole population to sustain the photocurrent. This extended surface-hole residence time favors net filling of SCL traps by electrons reaching the near-surface region, generating a larger trapped-electron population that can mediate recombination loss. In contrast, glycerol oxidation accelerates interfacial hole consumption, reducing both the density and lifetime of accumulated surface holes and shifting the kinetic competition away from SCL trap filling and recombination loss. To test whether this interpretation is kinetically self-consistent, we implemented a minimal diffusion-kinetic model (see Note S2.5). Increasing the effective interfacial hole consumption rate constant (*k*_cons_) decreases the density and lifetime of the surface holes (Figure 4g), which delays the modeled buildup of trapped electrons in the SCL (Figure 4d). In this model, FTO-side excitation naturally contributes to the delayed onset of SCL trap filling since electrons generated near the FTO/BiVO_4_ interface must first reach the near-surface SCL region before they can be trapped there. The model is intentionally phenomenological and is used here as a mechanistic consistency check, rather than a quantitative fit. It accounts for the observed TR-PPPC trends without requiring changes in the intrinsic trap-state density or the initial trapping step. Since the CW-PPPC and TR-PPPC measurements were performed under higher-intensity excitation, the absolute trapped-electron response should not be interpreted as a direct measure of trap occupancy under one-sun conditions. Higher accumulated surface-hole densities may modify interfacial band bending, enhancing SCL trap filling and recombination losses.^24,50^ Accordingly, we use PPPC as a mechanistic probe of relative trapped-electron population dynamics under matched conditions rather than as a direct quantitative measure of trap occupancy under one-sun operating conditions.

To test how the applied potential modulates this microsecond trap-filling behavior within the mechanistic picture inferred above, we further analyze potential-dependent Δ*J*_IR_/*J*_vis_ transients (Figure 4f,h). For quantitative comparison, we extract the time constant of the slowest component from a phenomenological multiexponential rise fit and use it as an effective rise time for the microsecond buildup of the SCL trapped-electron population, rather than as an elementary electron-trapping rate (see Note S2.17). At 0.6 V_RHE_, the effective rise times are similar without and with glycerol (∼9.0 and ∼11.0 μs, respectively). This similarity suggests that, at low potential, the buildup is governed mainly by the time required for electrons generated under FTO-side excitation to reach trap states within the SCL and become trapped there. At this low potential, interfacial hole consumption remains inefficient, so the two electrolytes show similar SCL trap-filling kinetics, consistent with the limited glycerol-induced photocurrent improvement at low potentials (Figure 1a). Above 0.6 V_RHE_, the two electrolyte conditions show distinct potential dependences. In glycerol-free electrolyte, increasing the potential from 0.6 to 1.2 V_RHE_ causes only a modest change in the effective rise time, which remains nearly constant from ∼9.0 to ∼10.1 μs. This indicates that, during water oxidation, the microsecond SCL trap-filling dynamics remain largely unchanged. In contrast, with 1.0 M glycerol, the effective rise time increases from ∼ 11.0 to ∼ 19.5 μs over the same potential range, showing that the trapped-electron population buildup becomes progressively delayed. This potential-dependent delay is consistent with more positive potential enhancing glycerol-driven interfacial hole consumption, thereby shifting the kinetic balance away from SCL trapped-electron buildup.

Taken together, the product analysis and operando optical/photocurrent measurements establish a consistent kinetic picture of how glycerol oxidation reduces charge carrier losses in BiVO_4_ photoanodes. Rate-law analysis reveals a distinct apparent second-order hole consumption regime for glycerol oxidation relative to water oxidation. PIA/TPC measurements show that, relative to water oxidation, glycerol oxidation sustains a higher photocurrent with a substantially lower surface-hole density, consistent with an approximately 32-fold higher apparent per-hole TOF. The attenuated light-off cathodic transient in the presence of glycerol is consistent with a suppressed BER.

CW-PPPC measurements show a strongly reduced SCL trapped electron population under glycerol oxidation conditions. PPPC mapping further identifies localized trap-filled hot spots in the glycerol-free electrolyte, while glycerol addition suppresses localized SCL trapped-electron buildup and yields a more spatially uniform response. TR-PPPC further shows that glycerol delays and attenuates the later microsecond buildup of SCL trapped electrons, rather than measurably altering the initial local trapping. TA shows that glycerol does not measurably perturb the dominant early time bulk-hole dynamics up to the early microsecond regime, supporting the fact that early bulk processes are not the primary origin of the glycerol-induced kinetic differences observed here.

## Conclusions

This work identifies a kinetic design principle for mitigating charge carrier losses associated with SCL trap filling during PEC oxidation on BiVO_4_ photoanodes. In glycerol oxidation, the enhanced photocurrent arises from faster interfacial hole consumption, which shortens the residence time of surface holes and suppresses the microsecond buildup of trapped electrons in the SCL. Surface holes therefore play a dual role: they provide the oxidative equivalents required for interfacial oxidation but, when they persist at the interface, they also favor SCL trap filling and the associated recombination.

Recognizing this dual role refines the design target for catalyst, interface, and electrolyte engineering: productive interfacial hole transfer should be accelerated relative to the microsecond buildup of trapped electrons in the SCL, thereby limiting the associated recombination. More broadly, correlating operando optical observables with trap-selective photocurrent readouts provides a transferable strategy for identifying this kinetic window and guiding the design of photoelectrodes with an improved performance.

## Supplementary Material



## Notes

Experimental details (BiVO_4_ photoanode synthesis and physical characterization; PEC and IPCE measurements; HPLC product analysis; PIA, TPC, PPPC, and TA measurements; 365 nm LED one-sun-equivalent calibration; FE calculations for FA and DHA formation; conversion of PIA signals to surface-hole density and TOF; diffusion-kinetic model; additional XRD, UV–vis, SEM, and PEC data) (PDF)
