# Supplementary material for: Fast Interfacial Hole Consumption Suppresses Space–Charge Layer Trap Filling in BiVO4 Photoanodes
Source: J Am Chem Soc. 2026 Jun 19;148(25):26322–32. doi: 10.1021/jacs.6c05776 (PMC13339167; doi:10.1021/jacs.6c05776)
Supplement: Supplementary file 1 [file ja6c05776_si_001.pdf]

Supporting Information

for

Fast Interfacial Hole Consumption Suppresses Space-Charge Layer Trap Filling in $BiVO_4$ Photoanodes

Longren Li[1], Tong Wang[1], Beier Hu[1], Louise I. Oldham[1], Qiyun Chen[1], Keming Li[1], Chuxi Yang[1], Paransa Alimard[1], Zhu Meng[1], Haoqing Ning[1], James R. Durrant[2], Andreas Kafizas[1,3]*, Artem A. Bakulin[1]*

[1]*Department of Chemistry, Molecular Science Research Hub, 82 Wood Lane, White City Campus, Imperial College London, London, W12 0BZ, U.K.*

[2]*Chemistry Research Laboratory, University of Oxford, Oxford OX1 3TA, U.K.*

[3]*London Centre for Nanotechnology, South Kensington Campus, Imperial College London, London, SW7 2AZ, U.K.*

**Corresponding authors: a.kafizas@imperial.ac.uk; a.bakulin@imperial.ac.uk*

Table of Contents

Section S1. Experimental Details

S1.1 $BiVO_4$ photoanode preparation (via AA-CVD)

S1.2 Structural and optical characterization

S1.3 Photoelectrochemical (PEC) measurements

S1.4 Incident-photon-to-current conversion efficiency (IPCE) measurements

S1.5 Product analysis via high-performance liquid chromatography (HPLC)

S1.6 Photo-induced absorption (PIA) spectroscopy and transient photocurrent (TPC) measurements

S1.7 Pump-push photocurrent spectroscopy (PPPC)

S1.8 Transient absorption (TA) spectroscopy

Section S2. Supporting Information Notes

Note S2.1 Calibration of the 365 nm LED to a one-sun-equivalent photon flux

Note S2.2 Faradaic efficiency calculations for FA and DHA

Note S2.3 Conversion of PIA (550 nm) to surface hole density and photocurrent to electron flux

Note S2.4 Per-hole turnover frequency (TOF) extraction

Note S2.5 Diffusion-kinetic model

Note S2.6 X-ray diffraction confirmation of phase-pure monoclinic $BiVO_4$ films

Note S2.7 UV-vis absorbance and transmittance of $BiVO_4$/FTO

Note S2.8 Morphology and thickness of $BiVO_4$ films

Note S2.9 Dark electrochemical current background

Note S2.10 Current density-potential curves under AM 1.5G illumination

Note S2.11 Glycerol concentration dependence

Note S2.12 Photocurrent stability under continuous PEC operation

Note S2.13 Chopped-illumination *J-V* reveals light-off cathodic transients

Note S2.14 Push-intensity dependence of the PPPC response ΔJ_{IR}

Note S2.15 Pump-intensity-dependent experiments and raw ΔJ_{IR} maps

Note S2.16 Additional hole scavengers also suppress the trapped-electron population in the SCL

Note S2.17 Normalized time-resolved pump-push photocurrent (TR-PPPC) kinetics and fitting method

Note S2.18 Photocurrent-Intensity Dependence under High-Excitation Conditions

Note S2.19 Scavenger-control measurements and representative TA spectra

References

Section S1. Experimental Details

S1.1 $BiVO_4$ photoanode preparation (via AA-CVD)

Synthesis. Dense $BiVO_4$ films were deposited on fluorine-doped tin oxide (FTO) substrates (TEC 15, 2.5×1.3 cm, Pilkington NSG) by aerosol-assisted chemical vapor deposition (AA-CVD) method.[1-3] Vanadium acetylacetonate ($V(acac)_3$, 0.0732 g, Sigma-Aldrich), and triphenyl bismuth ($Bi(Ph)_3$, 0.0881 g, VWR Chemicals) were dissolved in 40 mL of a mixed solvent of acetone/methanol (3:1 v/v) to form the precursor solution. The precursor solution was converted into an aerosol using an ultrasonic nebulizer and delivered to the reactor using compressed air as a carrier gas (4 L/min). The aerosol stream flowed over the FTO substrate placed on a heated substrate holder at 400 °C, leading to thermal decomposition and deposition of $BiVO_4$. After deposition, the films were annealed in air at 500 °C for 12 h to improve crystallinity. The resulting $BiVO_4$ film thickness was ~550 nm, determined from cross-sectional SEM images.

Substrate cleaning. Prior to deposition, FTO substrates were cleaned by sequential sonication (10 min each) in detergent solution, deionized water, acetone, methanol, and deionized water, followed by drying under a nitrogen stream.

S1.2 Structural and optical characterization

X-ray diffraction (XRD). XRD patterns were recorded using a modified Bruker D2 diffractometer equipped with a LYNXEYE PSD silicon strip detector. Cu Kα radiation was used ($K\alpha_1$ λ = 1.54056 Å, $K\alpha_2$ λ = 1.54439 Å; intensity ratio 2:1). All samples were scanned from 15° to 60° in 2θ with an angular increment of 0.03°.

UV-vis spectroscopy. Absorption and transmission spectra were acquired using a PerkinElmer Lambda 25 UV-vis spectrophotometer (slit width: 5 nm) over 350-1000 nm.

Scanning electron microscopy (SEM). SEM images were obtained using a Zeiss Gemini Sigma 300 field-emission SEM operated at an acceleration voltage of 5 kV. An InLens detector was used with a working distance of 5-7 mm. Prior to imaging, samples were coated with ~15 nm Cr to reduce charging.

S1.3 Photoelectrochemical (PEC) measurements

All photoelectrochemical measurements were conducted in a custom "cappuccino" cell in a three-electrode configuration using an Autolab potentiostat (PGSTAT101, Metrohm) controlled with Nova

software. $BiVO_4$/FTO served as the working electrode, a Pt mesh as the counter electrode, and an Ag/AgCl reference electrode (saturated KCl) as the reference electrode. The exposed geometric working area was defined by the cell aperture (S = 0.5 cm^2). All measurements used backside illumination (through the FTO side). A 365 nm LED was used for monochromatic measurements and calibrated to a one-sun-equivalent incident photon flux (**Note S2.1**). For white-light AM 1.5G illumination experiments, a 75 W xenon lamp (Hamamatsu) equipped with a KG3 filter was used.

The electrolyte was 0.5 M Na_2SO_4 (VWR Chemicals) prepared in deionized water (resistivity: 18.2 MΩ·cm). The pH was adjusted to pH 6 using H_2SO_4 and NaOH. This pH 6 was selected as a mildly acidic/near-neutral condition that balances glycerol-enhanced PEC response with $BiVO_4$ stability. For glycerol oxidation experiments, glycerol (≥99.5 %, Sigma-Aldrich) was added to a concentration of 1.0 M (unless otherwise stated). Potentials are reported versus the reversible hydrogen electrode (RHE) and were converted using the Nernst equation:

$$E_{RHE} = E_{Ag/AgCl} + E^0_{Ag/AgCl} + 0.0591 \times pH \qquad (Eq. 1)$$

where, $E_{Ag/AgCl}$ is the applied potential against Ag/AgCl reference electrode, and $E^0_{Ag/AgCl}$ is the standard potential of the reference electrode at 25 °C (0.197 V).

J-V scans (linear sweep voltammetry, LSV) were acquired from negative to positive potentials at a scan rate of 10 mV s^{-1}. Dark scans were recorded under otherwise identical conditions. Chopped-light chronoamperometry (CA) was recorded at 1.23 V_{RHE} under the same conditions using one-sun-equivalent 365 nm LED illumination with a 20 s light-on/20 s light-off duty cycle.

S1.4 Incident-photon-to-current conversion efficiency (IPCE) measurements

IPCE spectra were measured at 1.23 V_{RHE} under backside illumination. Monochromatic light (300-600 nm) was generated using a xenon lamp coupled to a monochromator (OBB-2001, Photon Technology International). The incident power at each wavelength was measured at the sample position using an optical power meter (PM100D, Thorlabs) equipped with a calibrated UV power sensor (S120UV, Thorlabs). The photocurrent response was recorded using an Autolab potentiostat (PGSTAT101, Metrohm) controlled by Nova software. The IPCE was calculated as follows:

$$IPCE = \frac{1239.8 \times I_{ph}}{\lambda \times P_{mono}} \times 100\% \qquad (Eq. 2)$$

where, I_{ph} (mA cm^{-2}) is the photocurrent density, P_{mono} (mW cm^{-2}) is the monochromatic light power

density at the sample plane, and λ (nm) is the incident light wavelength.

S1.5 Product analysis via high-performance liquid chromatography (HPLC)

After 2 h of PEC operation under one-sun-equivalent 365 nm LED illumination at the indicated potential, the electrolyte was collected and analyzed by high-performance liquid chromatography (HPLC). Analyses were performed using an Agilent 1260 Infinity system equipped with a diode-array detector (DAD). Separation was achieved using a Hi-Plex H column (PL1F70-6830). The mobile phase consisted of 5 mM H_2SO_4 (aq)/acetonitrile (HPLC grade) in a volumetric ratio of 70:30 (v/v) at a flow rate of 0.4 mL min^{-1}. The electrolyte was filtered through a hydrophilic PTFE filter (0.22 μm) prior to analysis. The injection volume was 20 μL, and UV absorbance was monitored at 195 nm. Total Faradaic efficiency (FE) calculations are described in **Note S2.2**.

S1.6 Photo-induced absorption (PIA) spectroscopy and transient photocurrent (TPC) measurements

Photoinduced absorption (PIA) spectroscopy was implemented in a pump-probe configuration like that reported previously.[4] The PIA signal was monitored at 550 nm as an operando proxy for long-lived holes accumulated at/near the $BiVO_4$ surface and was recorded simultaneously with transient photocurrent (TPC) during 365 nm LED excitation. Measurements were performed in a three-electrode cappuccino cell and electrolyte configuration described in **Section S1.3.**

The probe beam was provided by a tungsten lamp (Bentham IL1) and spectrally selected at 550 nm using a monochromator. The transmitted probe intensity was detected using a Si photodiode (Hamamatsu S3071) and digitized using a data-acquisition (DAQ) card (National Instruments, NI USB-6211). A 530 nm long-pass filter (Comar Instruments) was placed in the detection path to suppress scattered 365 nm pump light and short-wavelength stray light. Photoexcitation was provided by a 365 nm UV LED operated as a square pulse (30 s light-on; total acquisition time, 90 s). The LED was gated using a MOSFET (STF8NM50N, STMicroelectronics) driven by a function generator (TG300, Thurlby Thandar Instruments), and the LED current was supplied by a power supply (QL564P, TTi). Data acquisition was controlled using a custom LabVIEW software.

The PIA response was reported as the change in optical density at 550 nm ($\Delta OD_{550\ nm}$), calculated from the change in probe transmittance relative to the dark baseline (i.e., decreased transmittance corresponds to a positive $\Delta OD_{550\ nm}$). Conversion of $\Delta OD_{550\ nm}$ to surface hole density (p_{surf}) and conversion of photocurrent density to extracted electron flux (J) are described in **Note S2.3**.

S1.7 Pump-push photocurrent spectroscopy (PPPC)

All spectroscopic measurements in this study were performed under backside illumination through the FTO side to maintain a consistent carrier-generation profile and a stable optical geometry. In the front-illumination geometry, the optical beams would pass through the electrolyte before reaching the $BiVO_4$ film, which can introduce scattering, refraction, pulse dispersion, and beam-overlap instability, particularly for pulsed TR-PPPC/TA measurements and weak PPPC/TA signals.

Continuous-wave pump-push photocurrent (CW-PPPC). CW-PPPC measurements were performed using two co-aligned continuous-wave (CW) diode lasers (Thorlabs): a 405 nm pump (CPS405) and a 980 nm push (CPS980). Unless otherwise stated, the pump intensity is 0.14 W cm^{-2} and push intensity is 0.85 W cm^{-2}. $BiVO_4$ photoanodes were mounted in a custom photoelectrochemical (PEC) cell that enabled contact between the electrode and electrolyte through a circular aperture (diameter, 1 mm), reducing noise from the electrolyte. The pump and push beams were combined using a dichroic mirror and focused through the aperture onto the FTO side of the sample to a spot diameter of ~0.3 mm. To isolate the push-induced photocurrent, the pump beam was kept on continuously, while the push beam was modulated using an optical chopper system (Thorlabs MC2000B) at 717 Hz. The photocurrent component at the modulation frequency was recorded using a lock-in amplifier (Zurich Instruments MFLI) referenced to the chopper signal. Unless otherwise stated, measurements were conducted in a three-electrode configuration with a Pt mesh counter electrode and an Ag/AgCl reference electrode. The MFLI was also used as a constant-voltage source to apply the bias potential to the working electrode.

For the measurements shown in **Fig. 3c**, the pump intensities are 0.014, 0.028, 0.057, 0.085, 0.13, 0.14, 0.28, 0.42, 0.70, 1.4 W cm^{-2} in the glycerol-free electrolyte, and 0.14, 0.28, 0.42, 0.70, 1.4 W cm^{-2} in the electrolyte with 1.0 M glycerol.

Temperature control of the PEC cell was achieved using a custom-built proportional-integral-derivative (PID) feedback system. An Arduino microcontroller (Arduino Uno R3) was used to implement the PID algorithm and regulate the power delivered to a resistive heating element attached to the cell. The electrolyte temperature was monitored in real time using a digital temperature sensor (DHT11) positioned to probe the electrolyte temperature. The measured temperature was used as the PID feedback input to maintain the setpoint temperature during measurements.

CW-PPPC mapping. Spatially resolved CW-PPPC mapping was performed by raster scanning the sample under backside (FTO-side) illumination. The pump-push excitation and detection optics were

identical to those used for CW-PPPC measurements, except that the sample was mounted on a motorized translation stage (Standa 192796) controlled using a Standa motion controller (Standa 8SMC5). The sample was translated into a point-by-point raster pattern while recording the push-induced photocurrent at each position using lock-in detection. The effective optical spot diameter on the sample was ~120 μm. The scan step size and dwell time per pixel were 50 μm and 10 ms, respectively. Unless otherwise stated, the electrochemical configuration, electrolyte, biasing, and pump/push modulation conditions were the same as in the CW-PPPC measurements.

ns-μs TR-PPPC. Time-resolved PPPC (TR-PPPC) measurements in the ns-μs regime were carried out using pulsed pump and push excitation. The continuous-wave diode lasers used for CW-PPPC were replaced by a Ti:sapphire regenerative amplifier (Astrella, Coherent; 800 nm, 4 kHz) and an Nd:YAG laser (P1725, INNOLAS; 1064 nm, 4 kHz, ~3 ns). The 800 nm output from the Astrella was frequency-doubled in a β-barium borate (BBO) crystal to generate 400 nm pump pulses via second-harmonic generation, whereas the 1064 nm output from the Nd:YAG laser was used directly as the push pulse. Synchronization of the pump and push pulse trains, as well as control of the pump-push delay, was achieved using an electrical delay generator (DG645, Stanford Research Systems). The pump fluence is 7.71 μJ cm^{-2} and push fluence 1.56 mJ cm^{-2}.

The pump-induced and push-induced photocurrent components were recorded simultaneously using a two-channel lock-in amplifier (MFLI, Zurich Instruments), which also supplied the DC bias to the working electrode. The pump-induced photocurrent was detected on one channel referenced to the Astrella trigger output (4 kHz). The push-induced photocurrent was detected on the second channel by amplitude-modulating the 1064 nm push beam with an optical chopper (MC2000B, Thorlabs) at 717 Hz and using the chopper reference output for lock-in detection. To minimize electrical pickup noise under applied bias, the PEC cell, sample, and electrical connections were enclosed in a grounded Faraday cage.

Spatial overlap of the pump and push beams at the sample plane was optimized by maximizing their transmission through a 300 μm diameter pinhole positioned at the sample location. A focusing lens (f = 10 cm) was placed upstream of the PEC cell, and the sample was positioned approximately 11 cm from the lens to maximize the spatial overlap, as verified by the pinhole alignment procedure.

fs-ns TR-PPPC. TR-PPPC measurements in the fs-ns regime were performed using a 400 nm pump and a 1300 nm infrared push. The 400 nm pump pulses were generated by frequency-doubling the 800 nm output from the same Ti:sapphire regenerative amplifier (Astrella, Coherent; 800 nm, 4 kHz) in a BBO crystal. The pump beam was directed through a mechanical delay stage and modulated at 2 kHz using an optical chopper. The 1300 nm push pulses were generated using an optical parametric amplifier (TOPAS-

Prime, Coherent) pumped by the same Astrella source. The TR-PPPC signal was detected using a lock-in amplifier (MFLI, Zurich Instruments). TR-PPPC traces measured on different setups were stitched together by scaling adjacent traces to coincide within their overlapping delay regions, yielding a continuous trace spanning delay times from the femtosecond to microsecond regime.

S1.8 Transient absorption (TA) spectroscopy

Transient absorption (TA) spectroscopy was used to monitor the hole-population kinetics at 1.2 V_{RHE} under operando conditions. To span the full lifetime window from femtoseconds to seconds, three complementary TA setups were employed. TA traces measured on different setups were combined by scaling ΔA traces to coincide at an overlapping delay time (within the overlap between adjacent time windows), yielding a continuous ΔA(t) trace from fs to s delays.

fs-ps TA. Ultrafast TA in the 0.2 ps-6 ns delay window was measured using a commercial broadband pump-probe femtosecond transient absorption spectrometer (Helios, Spectra Physics/Newport). A 1 kHz Ti:sapphire regenerative amplifier (800 nm, ~100 fs pulse duration) served as the fundamental laser source. Part of the 800 nm output was directed to an optical parametric amplifier (TOPAS Prime, Spectra-Physics) and a frequency mixer (NirUVis, Light Conversion) to generate visible 400 nm pump pulse. The pump was modulated at 500 Hz using a mechanical chopper. The remaining 800 nm beam was routed through a mechanical delay stage (6 ns time window) into a sapphire to generate a white-light continuum probe. The probe was split into reference and signal arms; the measured spectra were normalized to the reference and averaged over multiple scans to improve the signal-to-noise ratio. Pump and probe were spatially overlapped on the sample with a 200 μm diameter beam. The pump fluence was kept at 127.3 μJ cm^{-2}.

ns-μs TA. Nanosecond-to-microsecond TA measurements were performed using a custom setup. The probe continuum (450–900 nm) was generated by focusing the fundamental output of a Yb:KGW laser (Pharos, Light Conversion; 1030 nm, ~200 fs, 10 kHz) into a sapphire crystal. The pump source was a 450 nm nanosecond pulsed laser diode system (NPL45C, Thorlabs) synchronized to the 10 kHz master clock; the minimum pump pulse width was 6 ns. The pump beam was mechanically modulated at 5 kHz (i.e., every other laser shot) using a chopper synchronized to the 10 kHz repetition rate. Pump and probe beams were spatially overlapped on the sample with an effective spot diameter of ~200 μm. The pump fluence was 12.7 μJ cm^{-2}.

μs-s TA. Slow TA kinetics from microsecond to second delays were measured using a flash-TA setup with a Nd:YAG excitation source (Big Sky Laser Technologies). The excitation pulse was delivered by

the third harmonic (355 nm) at a repetition rate of 0.33 Hz (pump fluence 100 μJ cm^{-2}) and guided to the sample via a 0.5 cm-diameter liquid light guide. The probe beam was provided by the same tungsten lamp with monochromator configuration used for operando PIA and set to 550 nm. A 530 nm long-pass filter was used to suppress scattered pump light and short-wavelength stray light reaching the detector. The signal was amplified (Costronics amplifier box) and recorded using a Tektronix TDS2012c oscilloscope (μs-ms range) and a National Instruments DAQ card (NI USB-6211; ms-s range). Data acquisition was operated via LabVIEW and processed in MATLAB.

Section S2. Supporting Information Notes

Note S2.1 Calibration of the 365 nm LED to a one-sun-equivalent photon flux

Monochromatic 365 nm LED illumination was used for PEC/PIA measurements. To report the 365 nm intensity on a "one-sun-equivalent" basis, we follow the photon-flux calibration procedure reported by Ma et al.,[5] in which the monochromatic photon flux is matched to the integrated AM 1.5G photon flux below the $BiVO_4$ absorption edge ($\lambda \leq 500$ nm). The calibration was performed as follows:

The AM 1.5G spectral photon flux, $\Phi(\lambda)$, is obtained from the AM 1.5G solar spectral irradiance, P(λ), via

$$\Phi(\lambda) = \frac{P(\lambda)}{E(\lambda)} = \frac{P(\lambda)\lambda}{hc} \qquad (Eq. S3)$$

where $\Phi(\lambda)$ is in m^{-2} s^{-1} nm^{-1}, $P(\lambda)$ is in W m^{-2} nm^{-1}, $E(\lambda) = hc/\lambda$ is the photon energy (J), h is Planck's constant, and c is the speed of light.

For $BiVO_4$ (absorption edge ≈ 500 nm), the integrated AM 1.5G photon flux below 500 nm is

$$\Phi_{\lambda<500nm} = \int_{280\ nm}^{500\ nm} \Phi(\lambda) d\lambda \qquad (Eq. S4)$$

where 280 nm is the lower wavelength limit of the AM 1.5G spectrum used for the integration.

The corresponding one-sun-equivalent 365 nm LED irradiance is then calculated by converting $\Phi_{\lambda<500nm}$ to a monochromatic power flux at 365 nm:

$$P_{365\ nm\ LED} = \Phi_{\lambda<500nm} \times E(365\ nm) = \Phi_{\lambda<500nm} \times \frac{hc}{365\ nm} \qquad (Eq. S5)$$

where $P_{365\ nm\ LED}$ is in W m^{-2}.

Note S2.2 Faradaic efficiency calculations for FA and DHA

Faradaic efficiency (FE) for product i was calculated from the quantified amount of product and the total charge passed:

$$FE_i = \frac{n_{e,i} \times F \times N_i}{Q_{\text{total}}} \qquad (Eq. S6)$$

where $n_{e,i}$ is the number of electrons transferred per molecule of product i, F is Faraday's constant, N_i is the number of moles of product formed, and Q_{total} is the total charge (C) passed the system during PEC reaction, obtained by integrating the current-time trace over the PEC operative period.

Product moles were obtained from measured concentrations in HPLC measurement:

$$N_i = C_i \times V_{electrolyte} \qquad (Eq. S7)$$

where C_i is the HPLC-quantified concentration, and $V_{\text{electrolyte}}$ is the electrolyte volume.

Electron stoichiometries used here were defined by the following net oxidation half-reactions (written for electron accounting):

DHA (C3 product):

$$C_3H_8O_3 \rightarrow C_3H_6O_3 + 2H^+ + 2e^-$$

thus $n_{e,\text{DHA}} = 2$ per mole of DHA.

FA (C1 product): assuming carbon-balanced formation of three FA from one glycerol:

$$C_3H_8O_3 + 3H_2O \rightarrow 3HCOOH + 8H^+ + 8e^-$$

thus $n_{e,\text{FA}} = 8/3$ per mole of FA.

Note S2.3 Conversion of PIA (550 nm) to surface hole density and photocurrent to electron flux

The quasi-steady-state photoinduced absorption (PIA) signal was monitored at 550 nm because previous transient-absorption studies of $BiVO_4$ photoanodes identified a broad visible absorption band of long-lived photogenerated holes extending over approximately 500-900 nm, with a maximum near 550 nm.[6] This assignment was established using scavenger and bias-dependent measurements: the signal increased in amplitude and lifetime in the presence of an electron scavenger, was strongly suppressed by a hole scavenger, and increased under anodic bias, consistent with enhanced survival of photogenerated holes. The long-lived component was further correlated with transient photocurrent measurements and assigned to accumulated holes involved in interfacial recombination and water-oxidation kinetics. Following this established $BiVO_4$ TA/PIA assignment, the 550 nm PIA signal (ΔOD_{550nm}) is used herein to study hole accumulation at/near the $BiVO_4$ surface. To enable quantitative comparisons and rate-law analysis, ΔOD_{550nm} was converted into an effective surface hole density following the PIA calibration methodology established by the Durrant group and used in subsequent $BiVO_4$ rate-law studies.[5, 7] The extinction coefficient of $BiVO_4$ photogenerated holes at 550 nm was determined by correlating the PIA amplitude with the surface-hole population obtained from simultaneously measured transient photocurrent responses. In this calibration, the light-off discharge current was integrated to estimate the charge associated with accumulated surface holes, which was then converted into a surface-accumulated hole population. Plotting the 550 nm PIA amplitude against this surface-accumulated hole population gave a linear calibration, whose slope yielded $\varepsilon_{h^+}(550nm) = 420 \pm 20$ M^{-1} cm^{-1}.

Following Ma et al., the Beer-Lambert relationship is written for a surface-accumulated population as:

$$\Delta \mathrm{OD}_{550nm} = \varepsilon_{h^+}(550nm) \times p \qquad (Eq. S8)$$

where $\varepsilon_{h^+}(550nm)$ (M^{-1} cm^{-1}) is the molar extinction coefficient of photogenerated holes in $BiVO_4$, p is an effective surface hole density term in M·cm, and $\varepsilon_{h^+}(550nm) = 420 \pm 20$ M^{-1} cm^{-1} for $BiVO_4$ holes.

Conversion to an areal number density (per geometric area) is:

$$p_{surf} = \frac{\Delta \mathrm{OD}_{550nm} \times N_A}{\varepsilon_{h^+}(550nm) \times 1000 \times 10^{14}} \qquad (Eq. S9)$$

where p_{surf} is the areal hole density (holes nm^{-2}), N_A is Avogadro's constant, the factor 1000 converts mol L^{-1} to mol mL^{-1}, and the factor 10^{14} converts cm^2 to nm^2. Unless otherwise stated, p_{surf} (holes nm^{-2}) is reported per geometric area; any roughness correction would introduce an overall scaling factor but does not affect reaction-order extraction.

The quasi-steady-state photocurrent density j (A cm^{-2}) was converted to an extracted electron flux per unit area J (electrons s^{-1} nm^{-2}) as:

$$J = \frac{j}{e \times 10^{14}} \qquad (Eq. S10)$$

where e is the elementary charge, and the factor 10^{14} converts cm^{-2} to nm^{-2}. Here j is taken as the quasi-steady-state (plateau) photocurrent under continuous illumination (excluding initial capacitive transients), such that J represents the net extracted electron flux under operando conditions.

Note S2.4 Per-hole turnover frequency (TOF) extraction

The per-hole turnover frequency TOF (s^{-1}) is defined as:

$$TOF = \frac{J}{p_{surf}} \quad (Eq. S11)$$

where J is the quasi-steady-state interfacial electron flux, and p_{surf} is the quasi-steady-state surface hole density. Here, J is used as an operational proxy for the interfacial hole-consumption flux under quasi-steady-state conditions. This treatment assumes that, under quasi-steady-state operation, the electron extraction flux is equal to the interfacial hole-consumption flux.

In this work, TOF values were extracted at 30 s after turning on the one-sun-equivalent 365 nm square pulse (**Fig. 2c,d**) at 1.23 V_{RHE}. Reported TOF values: ~3.5 s^{-1} (without glycerol) and ~113.2 s^{-1} (with 1.0 M glycerol).

Note S2.5 Diffusion-kinetic model: hole-consumption-controlled microsecond build-up of trapped-electron population in the SCL of $BiVO_4$

To test whether the experimentally inferred mechanism is kinetically self-consistent, we implemented a basic diffusion-kinetic model for a $BiVO_4$ film under backside excitation through the FTO side. The model is intentionally phenomenological and is used as a mechanistic consistency check rather than a quantitative fit. Its purpose is to formalize the inferred causal link between faster interfacial hole consumption, lower surface-hole accumulation, and suppressed SCL trap filling.

The $BiVO_4$ layer was treated as a one-dimensional slab of thickness L, with $x = 0$ at the FTO/$BiVO_4$ interface, and $x = L$ at the $BiVO_4$/electrolyte interface. Because excitation is introduced through the FTO side, the initial carrier generation profile was taken to decay from $x = 0$:

$$G_0(x) = G_{00}e^{-\alpha x} \qquad (Eq. S12)$$

where α is an effective absorption coefficient, G_{00} is a factor that sets the initial photocarrier generation amplitude at the FTO/$BiVO_4$ interface ($x = 0$). The electron and hole populations, $n(x,t)$ and $p(x,t)$, were then described by diffusion with phenomenological first-order bulk loss terms:

$$\frac{\partial n}{\partial t} = D_n \frac{\partial^2 n}{\partial x^2} - k_{\text{loss,e}}\, n(x,t) \qquad (Eq. S13)$$

$$\frac{\partial p}{\partial t} = D_p \frac{\partial^2 p}{\partial x^2} - k_{\text{loss,h}}\, p(x,t) \qquad (Eq. S14)$$

where D_n and D_p are effective diffusion coefficients and $k_{\text{loss},n}$ and $k_{\text{loss},p}$ are phenomenological bulk loss constants. To describe the long-lived interfacial hole population that controls both the residual TA response and the subsequent SCL trap filling, an effective interfacial hole population, $h_{\text{int}}(t)$, was introduced:

$$\frac{dh_{\text{int}}(t)}{dt} = k_{cap}\, p(L,t) - k_{cons}\, h_{\text{int}}(t) \qquad (Eq. S15)$$

where k_{cap} is an effective capture term and k_{cons} is the interfacial hole-consumption rate constant. Increasing k_{cons} therefore reduces both the magnitude and lifetime of the interfacial hole population, consistent with the experimentally observed suppression of persistent surface-hole accumulation in the presence of glycerol. The trapped-electron population associated with near-surface/SCL trap states, $n_{\text{tr}}(t)$, was then written as:

$$\frac{dn_{\text{tr}}(t)}{dt} = k_{trap}\, n(L,t)\, h_{\text{int}}(t) - k_{det}\, n_{\text{tr}}(t) \qquad (Eq. S16)$$

where k_{trap} is an effective trapping coefficient and k_{det} is a detrapping/decay constant. In this framework, FTO-side excitation naturally gives rise to a delayed onset of SCL trap filling because electrons must first reach the near-surface SCL region before they can be trapped there. The simulated TR-PPPC response was therefore taken to scale with the trapped-electron population, consistent with the delayed microsecond buildup observed experimentally.

To reflect the experimental observation that the 550 nm TA kinetics are essentially unchanged at early times and diverge only at longer delays, the simulated TA signal was represented phenomenologically as a bulk-dominated early-time term plus a smaller long-lived interfacial contribution,

$$S_{TA}(t) = A\, S_{bulk}(t) + B \int_0^L p(x,t)\ dx + C\, h_{\text{int}}(t) \qquad (Eq.S17)$$

where $S_{\text{bulk}}(t)$ is a phenomenological fast bulk-hole background and A, B, and C are weighting factors. In this construction, the TA response is dominated at early delay times by bulk holes, whereas the long-lived tail increasingly reflects the persistent interfacial hole population. This is consistent with the experimental TA data, in which glycerol does not measurably perturb the dominant early-time bulk hole dynamics but strongly suppresses the long-lived tail.

Within this minimal framework, increasing k_{cons} reduces and shortens the interfacial hole population and correspondingly delays the subsequent buildup of trapped-electron population in the SCL. The model therefore supports the mechanistic interpretation that faster interfacial hole consumption under glycerol oxidation lowers surface-hole accumulation and suppresses microsecond SCL trap filling. Because the model is deliberately minimal, it does not explicitly include interfacial electrostatics, band bending, or field-dependent drift, and should therefore be regarded as a phenomenological consistency check rather than a unique microscopic description.

Taken together, this minimal model is intended to reproduce the experimentally observed delay and suppression of the microsecond TR-PPPC signal under glycerol oxidation by increasing k_{cons}, without invoking a major change in the initial trapped-electron yield.

Table S1. Key parameters used in the diffusion-kinetic simulation.

Parameter	Value	Description
L	$5.5 \times 10^{-7}\ m$	$BiVO_4$ film thickness
α	$8.0 \times 10^{6}\ m^{-1}$	Effective absorption coefficient
D_n	$4.0 \times 10^{-8}\ m^2\ s^{-1}$	Electron diffusion coefficient
D_p	$3.0 \times 10^{-9}\ m^2\ s^{-1}$	Hole diffusion coefficient
$k_{loss,n}$	$1.0 \times 10^{5}\ s^{-1}$	Bulk electron loss rate
$k_{loss,p}$	$3.0 \times 10^{4}\ s^{-1}$	Bulk hole loss rate
k_{cap}	$5.0 \times 10^{4}\ s^{-1}$	Interfacial hole-capture rate
k_{trap}	$5.0 \times 10^{8}\ s^{-1}$	Trapped-electron formation coefficient
k_{det}	$3.0 \times 10^{3}\ s^{-1}$	Trapped-electron decay rate
k_{cons_water}	$3.0 \times 10^{3}\ s^{-1}$	Hole-consumption rate, water
$k_{cons\_glycerol}$	$4.0 \times 10^{4}\ s^{-1}$	Hole-consumption rate, glycerol

Note S2.6 X-ray diffraction confirmation of phase-pure monoclinic $BiVO_4$ films

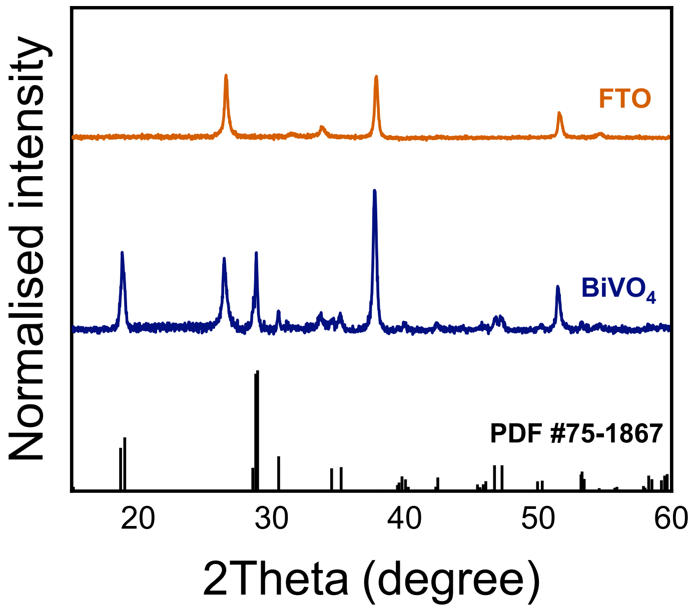

Figure S1. X-ray diffraction (XRD) patterns of the bare FTO substrate and AA-CVD produced $BiVO_4$ films on FTO, compared with the reference stick pattern for monoclinic scheelite $BiVO_4$ (PDF #75-1867). XRD was collected using Cu K_α radiation over 15-60° (2θ) with an angular increment of 0.03°. Peaks attributable to the FTO substrate are indicated for clarity; all remaining reflections are consistent with monoclinic $BiVO_4$, with no detectable impurity peaks.

Note S2.7 UV-vis absorbance and transmittance of $BiVO_4$/FTO

UV-vis absorbance and transmittance spectra were collected to characterize the optical window of the $BiVO_4$ films on FTO and to confirm minimal direct absorption in the NIR region relevant to the 980 nm "push" excitation used in PPPC. As shown in **Fig. S2**, $BiVO_4$ absorbs strongly below ~500 nm and becomes largely transparent at longer wavelengths, with high transmittance extending to 1000 nm. This supports that NIR illumination primarily perturbs the IR-reactivatable trapped-electron population rather than generating additional band-to-band carriers in $BiVO_4$ under the conditions used.

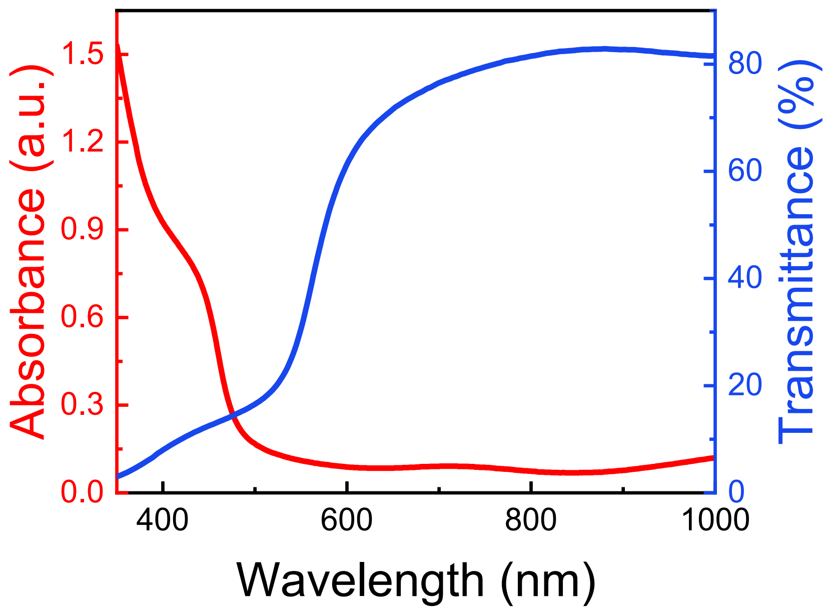

Figure S2. UV-vis absorbance (red, left axis) and transmittance (blue, right axis) spectra of $BiVO_4$ films on FTO over 350-1000 nm. Strong absorption is observed in the near-UV/blue region with a characteristic feature near ~440 nm, while absorbance is low and transmittance remains high for wavelengths >~500 nm, indicating minimal $BiVO_4$ absorption in the NIR region.

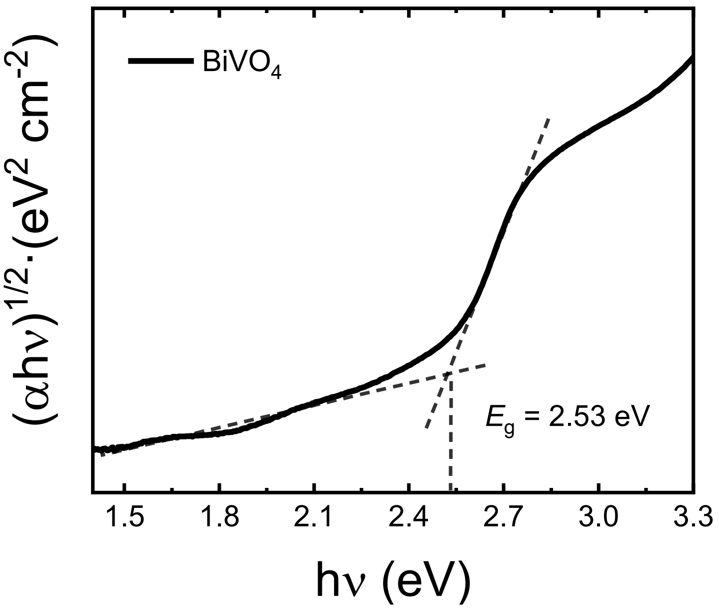

Figure S3. Tauc plot $(\alpha h\nu)^{1/2}$ versus photon energy $h\nu$ for the $BiVO_4$ film (indirect allowed transition). The dashed lines indicate the absorption edge and the extrapolation used to determine the optical band gap, giving $E_g = 2.53$ eV.

Note S2.8 Morphology and thickness of $BiVO_4$ films

Scanning electron microscopy (SEM) was used to evaluate the surface morphology and film continuity of the $BiVO_4$ photoanodes on FTO. Top-view images (**Fig. S4**) show a compact, granular morphology with closely packed grains and no obvious pinholes or large cracks over the imaged areas, consistent with a continuous light-absorbing layer suitable for operando optical and photoelectrochemical measurements. Cross-sectional SEM (**Fig. S5**) confirms a conformal $BiVO_4$ coating on FTO with an average thickness of ~550 nm (from multiple positions along the cross-section), which is the thickness used as the geometric film thickness in our minimal diffusion-kinetic analysis (**Note S2.5**).

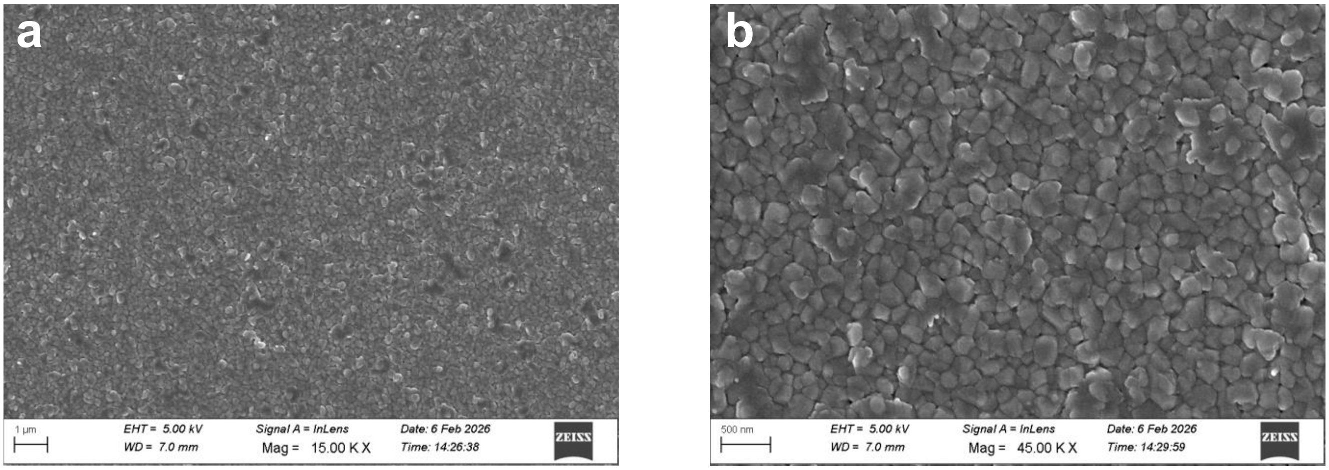

Figure S4. Top-view SEM images of the $BiVO_4$ film surface on FTO at two magnifications (a) 15k and (b) 45k, showing a compact granular morphology without obvious pinholes or large cracks in the imaged regions.

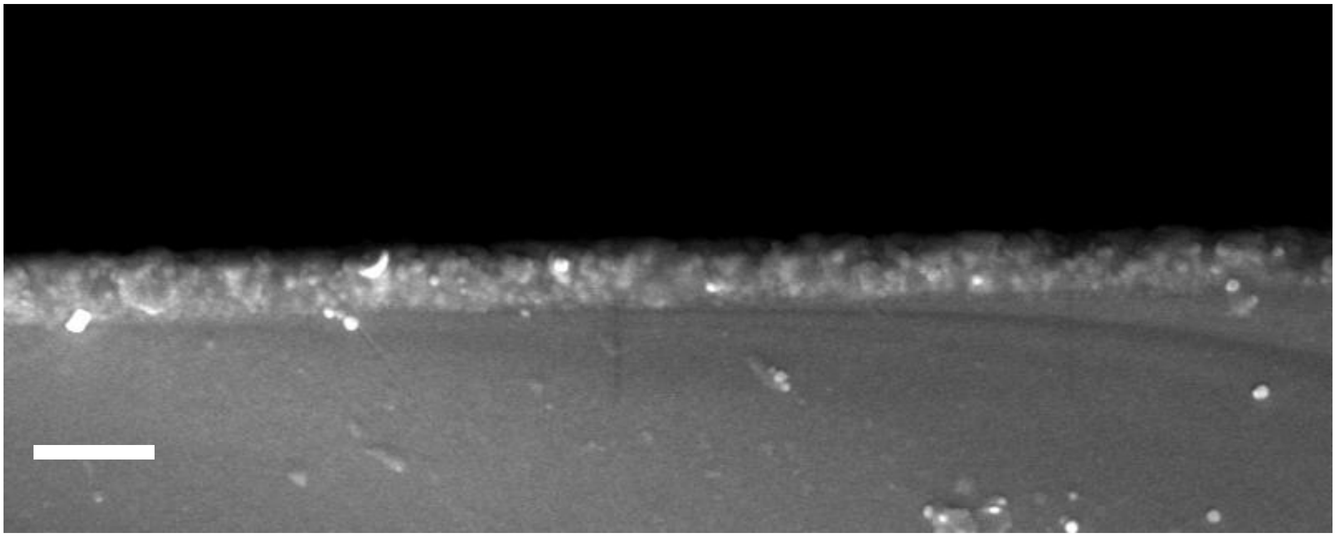

Figure S5. Cross-sectional SEM image of $BiVO_4$/FTO showing a continuous $BiVO_4$ layer with an average thickness of ~550 nm. Scale bar: 1 µm.

Note S2.9 Dark electrochemical current background

Dark current-potential (*J*-*V*) measurements were performed to quantify the electrochemical background of the $BiVO_4$/FTO electrodes. As shown in **Fig. S6**, both electrolytes (0.5 M Na_2SO_4, pH 6, with and without 1.0 M glycerol) exhibit negligible dark anodic current over the operational window, with a noticeable rise in dark current only at sufficiently positive bias (> ~2.0 V_{RHE}). The presence of glycerol slightly increases the magnitude of the dark anodic current at very high bias, but does not introduce substantial dark currents at or below the potential used for the operando kinetic analyses (e.g., 1.23 V_{RHE}).

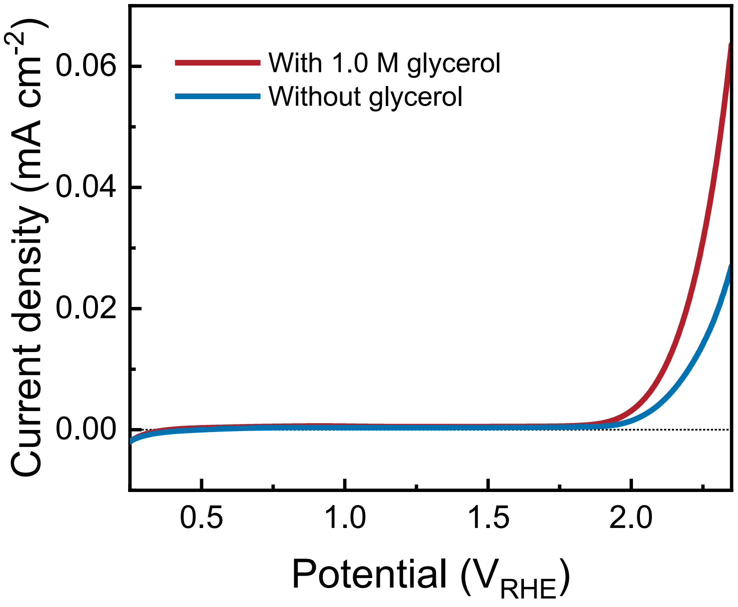

Figure S6. Dark *J*-*V* curves of $BiVO_4$ in 0.5 M Na_2SO_4 (pH 6) with and without 1.0 M glycerol over an extended potential window. Scan rate: 10 mV s^{-1}.

Note S2.10 Current density-potential curves under AM 1.5G illumination

To verify that the glycerol-induced PEC performance enhancement of $BiVO_4$ is not specific to the 365 nm illumination used for operando spectroscopy, we measured *J*-*V* curves under AM 1.5G illumination in the same 0.5 M Na_2SO_4 (pH 6) with and without 1.0 M glycerol. As shown in **Fig. S7**, glycerol consistently shifts the photocurrent onset cathodically and increases the photocurrent density across the measured potential range under AM 1.5G illumination. The corresponding dark currents remain negligible over this potential window, indicating that the observed differences arise from photo-driven processes rather than a substantial electrochemical background.

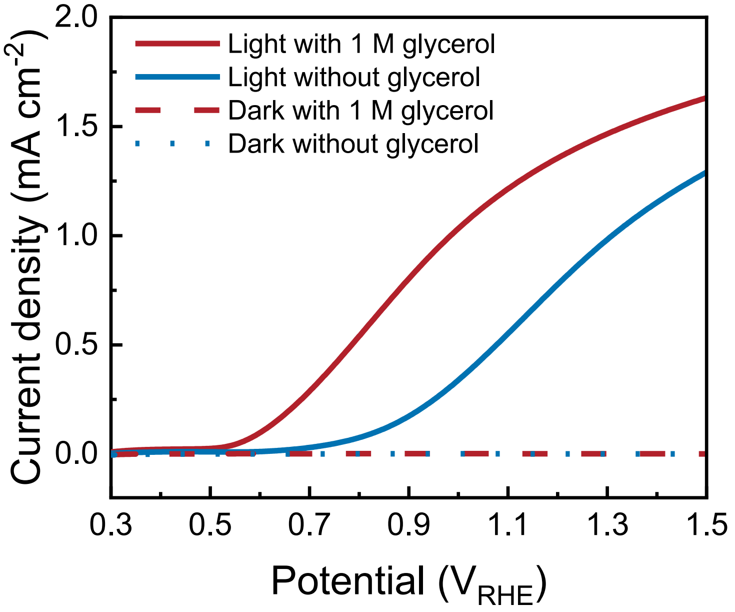

Figure S7. *J*-*V* curves of $BiVO_4$ under AM 1.5G back illumination in 0.5 M Na_2SO_4 (pH 6) with and without 1.0 M glycerol. Corresponding dark curves (dashed lines) are included for comparison and show negligible current over the same potential window. Scan rate: 10 mV s^{-1}.

Note S2.11 Glycerol concentration

To evaluate the dependence of the photoelectrochemical (PEC) response on glycerol concentration, current density-potential (*J-V*) curves were recorded under back illumination (AM 1.5G) in 0.5 M Na_2SO_4 (pH 6) with glycerol concentrations ranging from 0 to 1.5 M (**Figure S8**). Increasing glycerol concentration increases the anodic photocurrent density and shifts the photocurrent onset to less positive potentials. The incremental enhancement becomes modest at ≥1.0 M glycerol under the present conditions, consistent with 1.0 M being sufficient to approach the plateau response. The dark current remains close to zero over the same potential window, indicating that the observed concentration dependence primarily reflects photo-driven processes rather than a substantial electrochemical background.

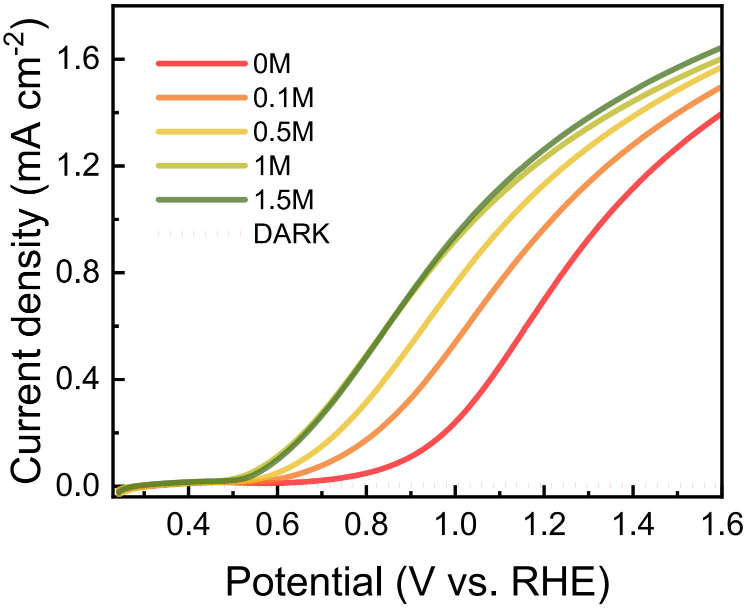

Figure S8. Current density-potential (*J-V*) curves of $BiVO_4$/FTO measured under AM 1.5G illumination (backside illumination through FTO) in 0.5 M Na_2SO_4 (pH 6) with added glycerol at 0, 0.1, 0.5, 1.0, and 1.5 M (as indicated). The dotted trace shows the dark response with 1.5 M glycerol measured under otherwise identical conditions. Scan rate: 10 mV s^{-1}.

Note S2.12 Photocurrent stability under continuous PEC operation

Photocurrent stability was evaluated by chronoamperometry at 1.23 V_{RHE} under continuous one-sun-equivalent 365 nm backside illumination in 0.5 M Na_2SO_4 (pH 6) with and without 1.0 M glycerol (**Fig. S9**). In both electrolytes, the photocurrent displays a rapid initial transient followed by a gradual decay over 2 h. The electrolyte with 1.0 M glycerol maintains a consistently higher photocurrent throughout the measurement, confirming that the glycerol-induced performance enhancement is sustained under extended operation. A short-lived current spike is observed at ~1 h for both traces; this feature coincides with deliberate electrolyte stirring and is therefore attributed to a transient hydrodynamic perturbation (e.g., temporary thinning of the diffusion layer and refresh of the local reactant/product environment at the electrode surface) rather than an irreversible change in the electrode. After stirring, the photocurrent returns to the same decay trend, indicating no evidence of catastrophic deactivation during the test window.

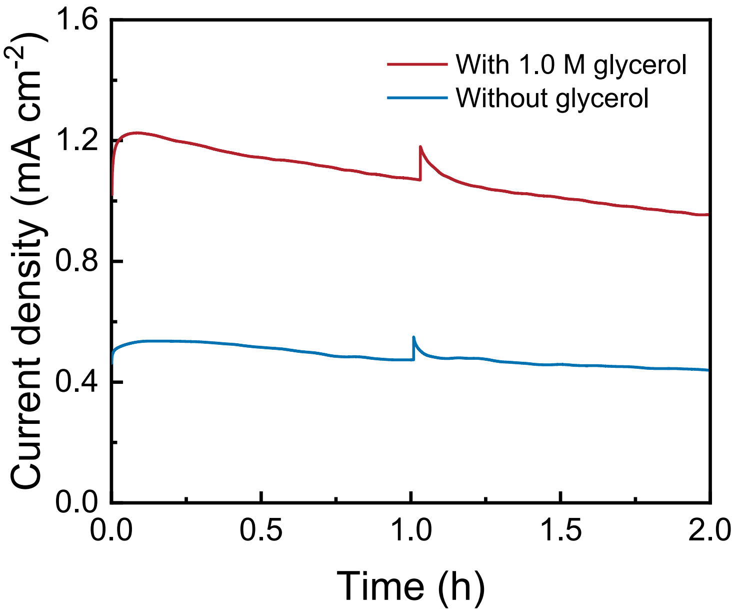

Figure S9. Photocurrent stability (chronoamperometry) measurements of $BiVO_4$ measured at 1.23 V_{RHE} under continuous one-sun-equivalent 365 nm backside illumination in 0.5 M Na_2SO_4 (pH 6) with and without 1.0 M glycerol. The transient spike at ~1 h corresponds to electrolyte stirring.

Note S2.13 Chopped-illumination *J-V* reveals light-off cathodic transients

Fig. S10 shows chopped-light *J-V* traces of $BiVO_4$ in 0.5 M Na_2SO_4 (pH 6) with and without 1.0 M glycerol. Panel a shows the full chopped-light current response, while panel b expands the near-zero current region to highlight the light-off cathodic transients. In glycerol-free electrolyte, the light-off cathodic transient persists over a broad potential range and is strongly attenuated only above ~1.3 V_{RHE}. In the presence of 1.0 M glycerol, this transient is strongly suppressed already above ~0.9 V_{RHE}, while the steady photocurrent is higher. These data provide the chopped-light reference used in the main text to compare light-off discharge behavior with the potential dependence of the CW-PPPC response.

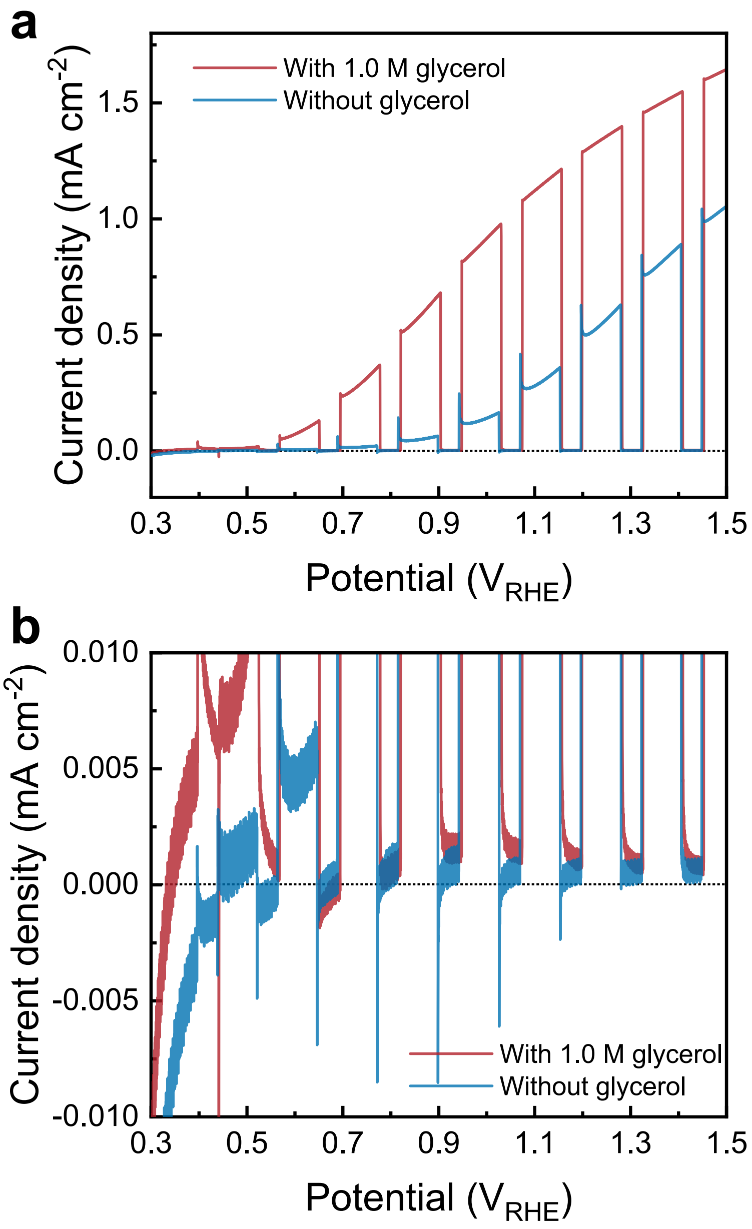

Figure S10. *J-V* curves of $BiVO_4$ under chopped one-sun-equivalent 365 nm LED back illumination (from FTO side) in 0.5 M Na_2SO_4 (pH 6) with and without 1.0 M glycerol.

Note S2.14 Push-intensity dependence of the PPPC response ΔJ_{IR}

We measured the push-intensity dependence of ΔJ_{IR} in the absence and presence of 1.0 M glycerol (**Fig. S11**). In both electrolyte conditions, ΔJ_{IR} increases approximately linearly with push intensity over the investigated range, indicating that the ΔJ_{IR} remains in the unsaturated regime under the conditions used here. The slope is lower in the presence of glycerol, consistent with a smaller trapped-electron population.

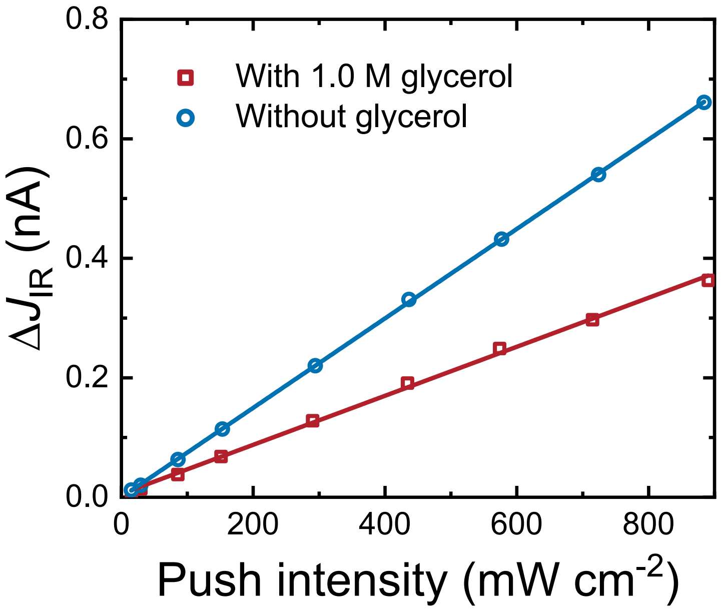

Figure S11. Push-intensity dependence of the CW-PPPC response, ΔJ_{IR}, measured in 0.5 M Na_2SO_4 (pH 6) with and without 1.0 M glycerol. The pump intensity is 0.15 W cm^{-2}. In both electrolyte conditions, ΔJ_{IR} shows an approximately linear dependence on push intensity over the investigated range, indicating that the PPPC readout was acquired in the linear, unsaturated regime. The reduced slope in the presence of glycerol is consistent with a weaker trapped-electron response.

Note S2.15 Pump-intensity-dependent experiments and raw ΔJ_{IR} maps

We measured the pump-intensity dependence of ΔJ_{IR} at the mesoscale trap-filled hot spot and a nearby normal region, while keeping the push intensity constant (**Fig. S12a**). ΔJ_{IR} increases with pump intensity at low excitation density and then reaches a plateau at higher pump intensity; this plateau is defined here as the saturated ΔJ_{IR} response, where additional pump power no longer appreciably increases ΔJ_{IR}. The trap-filled hot spot saturates at a higher ΔJ_{IR} value than the normal region.

The raw ΔJ_{IR} maps (**Fig. S12b,c**) show the same qualitative trend as the $\Delta J_{IR}/J_{vis}$ maps in **Fig. 3h,i**: without glycerol, the ΔJ_{IR} response remains stronger and spatially more inhomogeneous, whereas with 1.0 M glycerol it is more homogeneous and overall amplitude reduced.

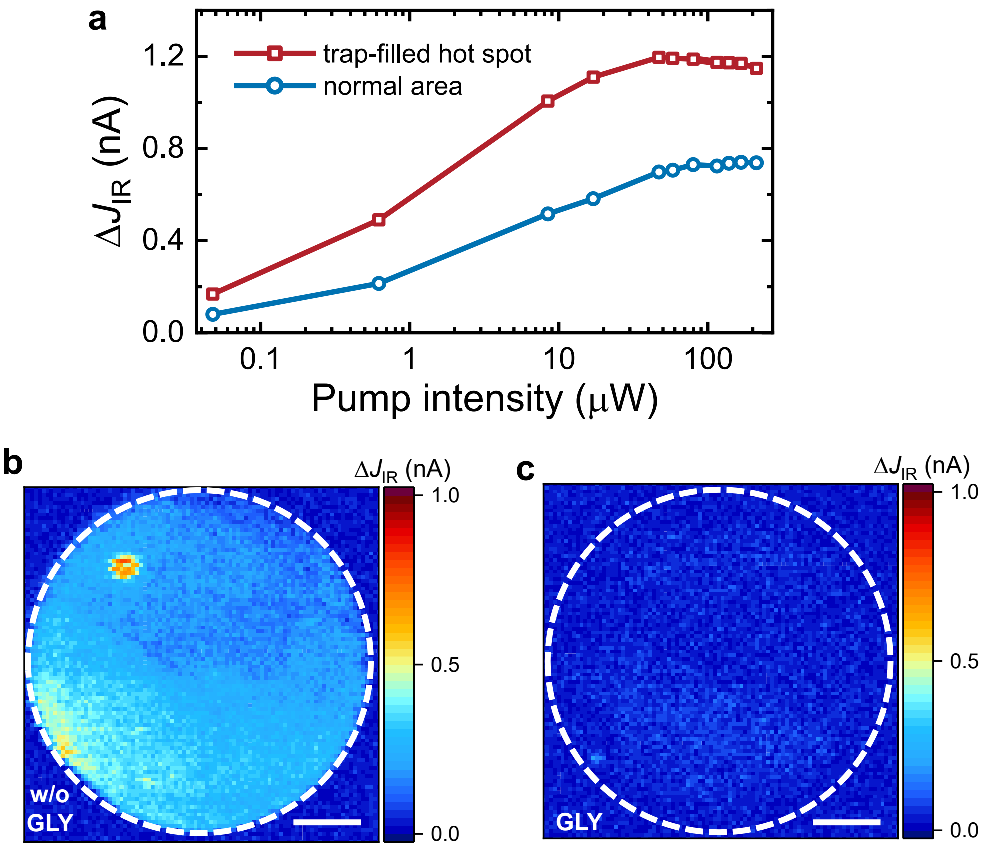

Figure S12. a) Pump-intensity dependence of ΔJ_{IR} measured under short-circuit conditions at the mesoscale trap-filled hot spot and a nearby normal region identified from the CW-PPPC map. The push intensity was kept constant while the pump intensity was varied. Spatial maps of the unnormalized ΔJ_{IR} measured at 1.23 V_{RHE} in 0.5 M Na_2SO_4 (pH 6). (b) without glycerol and (c) with 1.0 M glycerol, under otherwise identical conditions to **Fig. 3h,i**. The pump intensity is 0.14 W cm^{-2}, and the push intensity is 0.85 W cm^{-2}. White dashed circles delineate the illuminated/active area; scale bar, 1 mm; All measurements use backside (through the FTO side) illumination.

Note S2.16 Additional hole scavengers also suppress the trapped-electron population in the SCL

We measured spatially resolved ΔJ_{IR} maps in the presence of additional hole scavengers, under comparable CW-PPPC mapping conditions (**Fig. S13**). Relative to 0.5 M Na_2SO_4 (pH 6) without added scavenger (**Fig. S13a**), both 10% methanol (**Fig. S13c**) and 0.1 M Na_2SO_3 (**Fig. S13d**) strongly reduce ΔJ_{IR} across the mapped area, yielding weak and nearly featureless responses comparable to that observed with 1.0 M glycerol (**Fig. S13b**). The corresponding pixel-value distributions (**Fig. S13e,f**) confirm that all scavenger-containing electrolytes shift the ΔJ_{IR} response to low values, with **Fig. S13f** providing an expanded view of the low-ΔJ_{IR} range. These measurements are used as a qualitative control rather than a quantitative ranking of hole-scavenging kinetics, as the added scavengers differ in concentration and reaction chemistry.

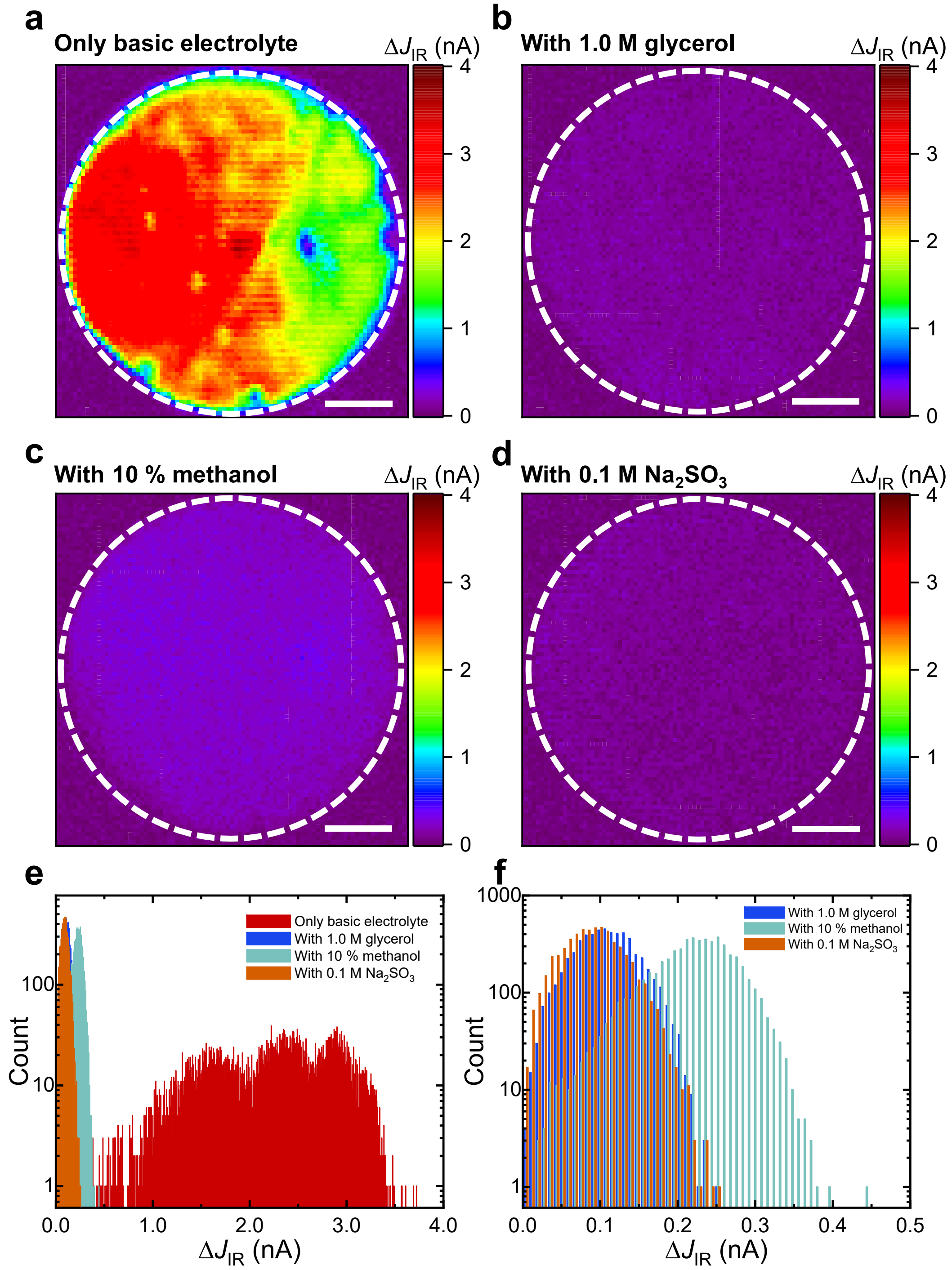

Figure S13. Spatial maps and pixel-value distributions of the ΔJ_{IR} measured at 1.23 V_{RHE} in 0.5 M Na_2SO_4 (pH 6) without added scavenger or with added hole scavengers. Spatial ΔJ_{IR} maps are shown for (a) electrolyte without added scavenger, (b) 1.0 M glycerol, (c) 10 % methanol, and (d) 0.1 M Na_2SO_3. (e) Corresponding ΔJ_{IR} pixel-value distributions for all four conditions. (f) Expanded view of the low-ΔJ_{IR} range for the scavenger-containing electrolytes, enabling clearer comparison among glycerol, methanol, and Na_2SO_3. The pump intensity is 0.14 W cm^{-2}, and the push intensity is 0.85 W cm^{-2}. White dashed circles delineate the illuminated/active area; scale bars, 1 mm. All measurements use backside illumination through the FTO side.

Note S2.17 Normalized time-resolved pump-push photocurrent (TR-PPPC) kinetics and fitting method.

The complete TR-PPPC kinetics were constructed by stitching together the fs-ns PPPC and ns-μs PPPC measurements. To compare the evolution of the TR-PPPC response independent of absolute signal amplitude, the ΔJ_{IR} traces in **Fig. S14a** were normalized to their values at the longest pump-push delay, whereas those in **Fig. S14b** were normalized to ΔJ_{IR} at a pump-push delay of 1 μs. This normalization is used only to compare relative SCL trapped-electron buildup kinetics.

Fitting analysis of the normalized TR-PPPC kinetics. The TR-PPPC traces were fitted using a phenomenological multiexponential rise function:

$$y_{fit}(t) = y_0 + \sum_i A_i \left(1 - e^{-\frac{t}{\tau_i}}\right) \qquad (Eq. S18)$$

where y_0 is an offset, and A_i and τ_i are the amplitudes and characteristic rise times of the individual components, respectively. This analysis is intended to parameterize the temporal evolution of the TR-PPPC response rather than to assign each component to a unique elementary process.

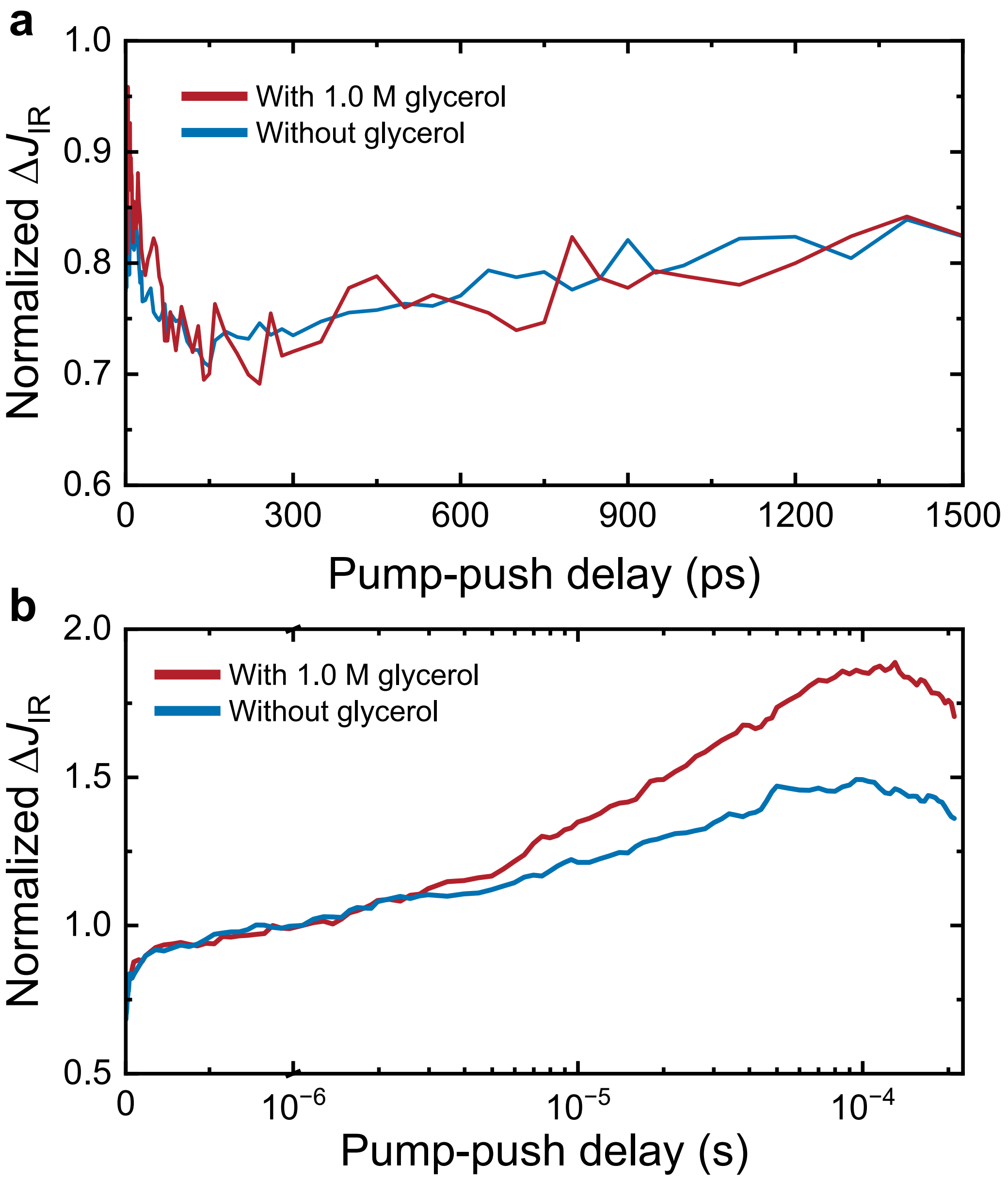

Figure S14. Normalized time-resolved pump-push photocurrent (TR-PPPC) responses for $BiVO_4$ photoanodes measured in 0.5 M Na_2SO_4 (pH 6) with and without 1.0 M glycerol (same conditions as **Fig. 4a**). (a) fs-ps TR-PPPC traces normalized to ΔJ_{IR} at the longest pump-push delay. (b) ns-μs TR-PPPC traces normalized to ΔJ_{IR} at a pump-push delay of 1 μs. The normalized representation facilitates comparison of the delay-dependent evolution of the TR-PPPC response under the two electrolyte conditions.

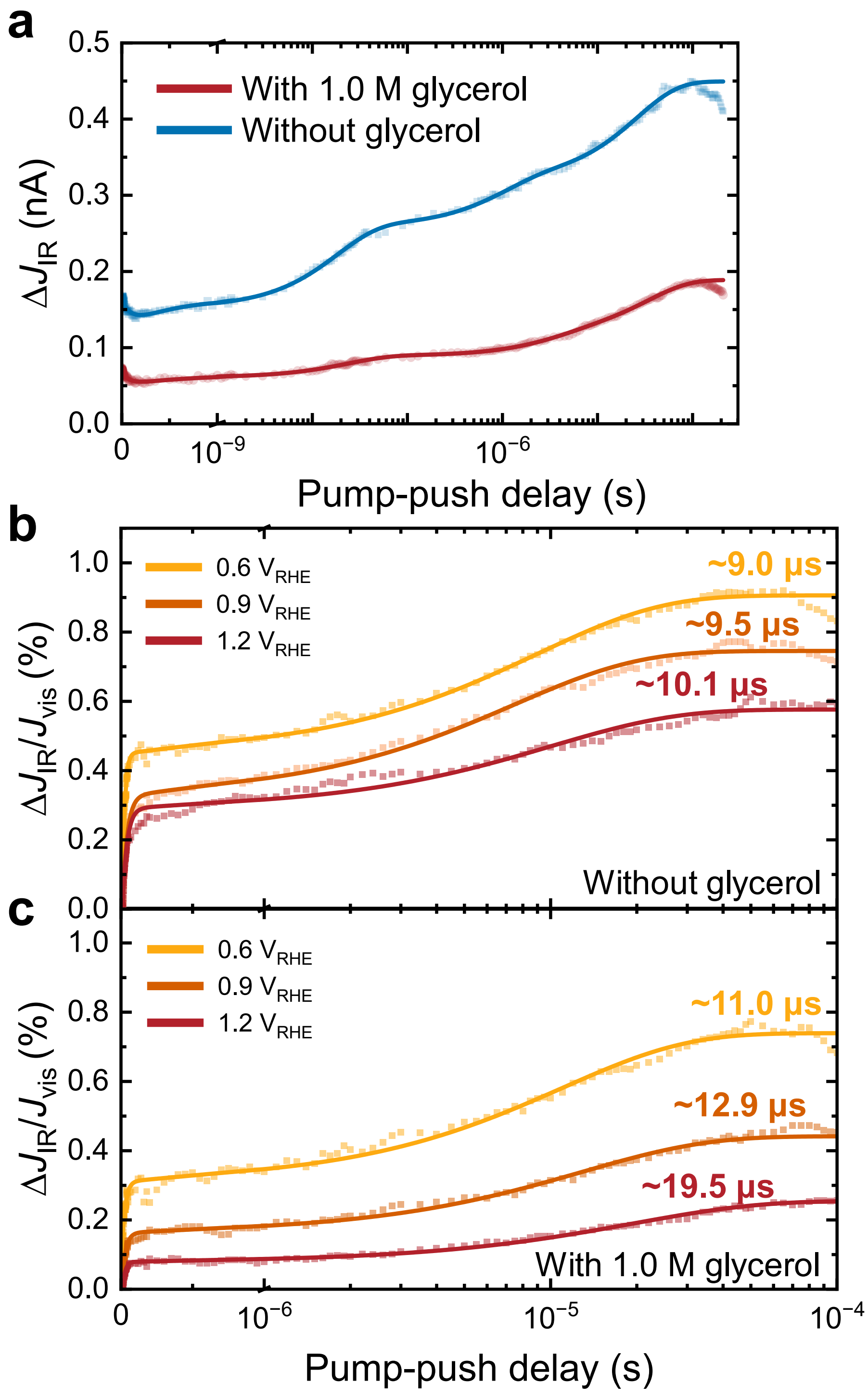

Figure S15. Time-resolved pump-push photocurrent (TR-PPPC) responses of $BiVO_4$ photoanodes measured in 0.5 M Na_2SO_4 (pH 6) in the absence and presence of 1.0 M glycerol. (a) Representative ΔJ_{IR} traces comparing the responses measured with and without 1.0 M glycerol. (b,c) $\Delta J_{IR}/J_{vis}$ traces, measured at 0.6, 0.9, and 1.2 V_{RHE} without glycerol (b) and with 1.0 M glycerol (c). Symbols represent experimental data, and solid lines are exponential fits used to extract the effective rise times annotated in each panel. All measurements were performed under the same conditions as in **Fig. 4a,g,h**.

Note S2.18 Photocurrent-Intensity Dependence under High-Excitation Conditions

To assess whether the excitation regime used in the PPPC measurements remained within the linear photocurrent regime, we measured the photocurrent as a function of pump intensity at 1.2 V_{RHE}. The photocurrent shows a clear sublinear dependence on pump intensity and is well described by a power-law relation, $J_{\mathrm{photo}} = J_0\varphi^{\gamma}$, with $\gamma = 0.61$. This deviation from linearity ($\gamma = 1$) indicates that, under the higher-intensity conditions used for PPPC, the photoresponse is no longer in the linear photocurrent regime. Accordingly, the high-intensity PPPC measurements are interpreted mechanistically as an amplified readout of trapped-electron dynamics, rather than as a quantitative reproduction of one-sun operating photocurrents.

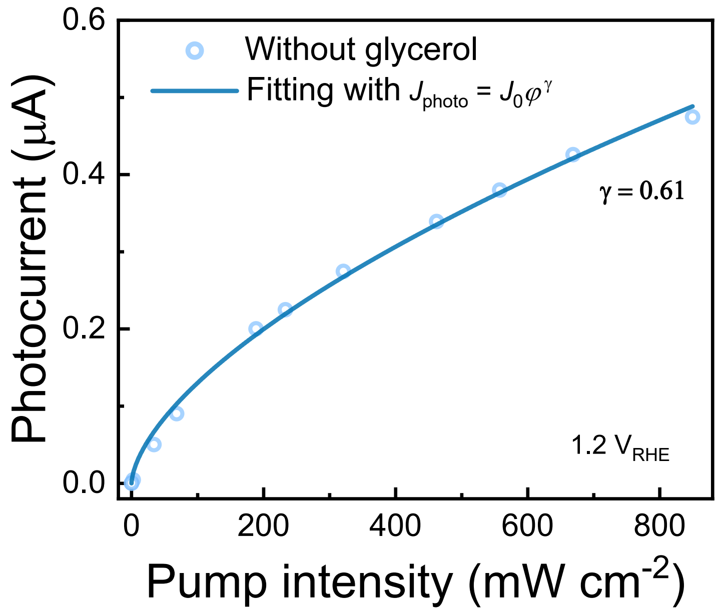

Figure S16. Photocurrent as a function of pump intensity for the $BiVO_4$ photoanode measured at ~1.2 V_{RHE} in 0.5 M Na_2SO_4 (pH 6). The data show a sublinear dependence on pump intensity and were fitted with $J_{\mathrm{photo}} = J_0\varphi^{\gamma}$.

Note S2.19 Scavenger-control measurements and representative TA spectra

Scavenger-control TA measurements were performed to further assess the assignment of the 550 nm TA signal. $BiVO_4$ was measured under otherwise identical conditions without added scavenger, with 6 mM $AgNO_3$ as an electron scavenger, and with 0.1 M Na_2SO_3 as a hole scavenger. Addition of $AgNO_3$ produces no substantial change in the 550 nm kinetics within the present measurement window, whereas Na_2SO_3 markedly accelerates the signal decay. This contrast supports the use of the 550 nm response as a primarily hole-sensitive probe under the present conditions, consistent with previous $BiVO_4$ TA assignments.[5-7]

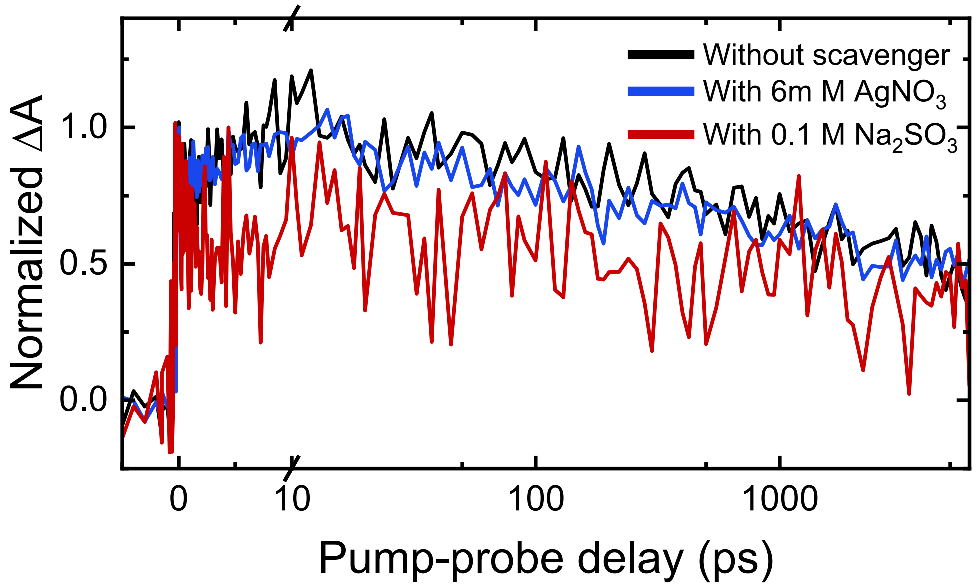

Figure S17. Scavenger-control TA kinetics of $BiVO_4$ monitored at 550 nm under otherwise identical conditions without added scavenger, with 6 mM $AgNO_3$ as an electron scavenger, and with 0.1 M Na_2SO_3 as a hole scavenger.

Representative broadband TA spectra corresponding to the **Fig. 4e** kinetics are shown in **Fig. S18** and **Fig. S19** for selected pump-probe delays in the fs-ps and ns-µs time windows, respectively. These spectra provide the spectral information for the 550 nm kinetic traces used in the main text. Across both the fs-ps and ns-µs time windows, the broadband TA spectra measured with and without 1.0 M glycerol show very similar spectral shapes and amplitudes (**Figs. S18** and **S19**). This indicates that glycerol does not measurably perturb the dominant early-time bulk carrier dynamics probed by TA. Although glycerol oxidation provides a faster interfacial hole-consumption pathway than water oxidation, this interfacial reaction remains slow relative to the ultrafast and early microsecond photophysical processes that determine the spectral profiles in **Figs. S18** and **S19**.

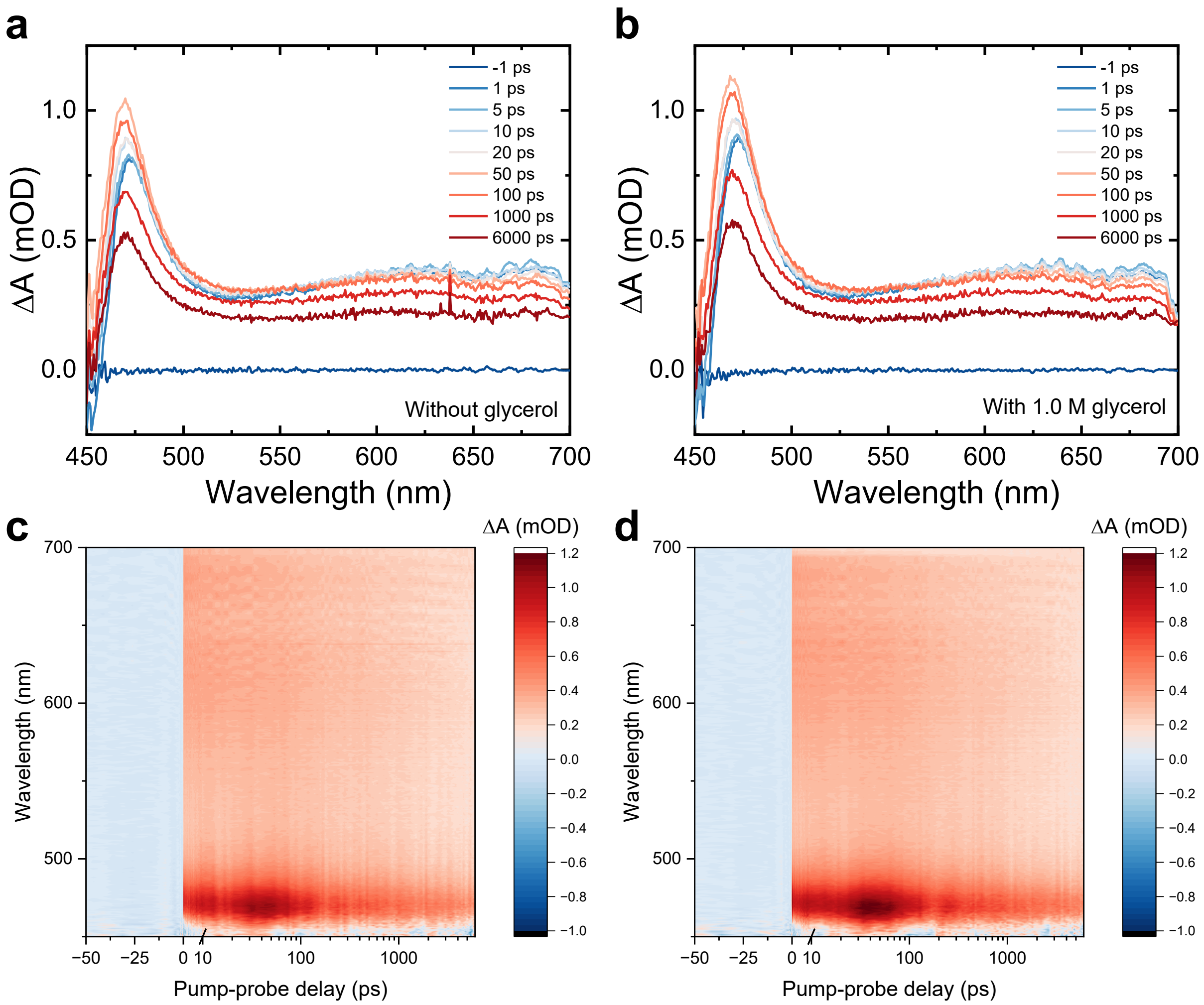

Figure S18. Representative fs-ps transient absorption (TA) spectra and spectral-temporal maps of $BiVO_4$ measured under the same operando electrolyte conditions as the 550 nm kinetics shown in **Fig. 4e**. (a,b) TA spectra at selected pump-probe delays from -1 ps to 6000 ps measured in 0.5 M Na_2SO_4 (pH 6) (a) without glycerol and (b) with 1.0 M glycerol. (c,d) Corresponding spectral-temporal TA maps for $BiVO_4$ measured (c) without glycerol and (d) with 1.0 M glycerol.

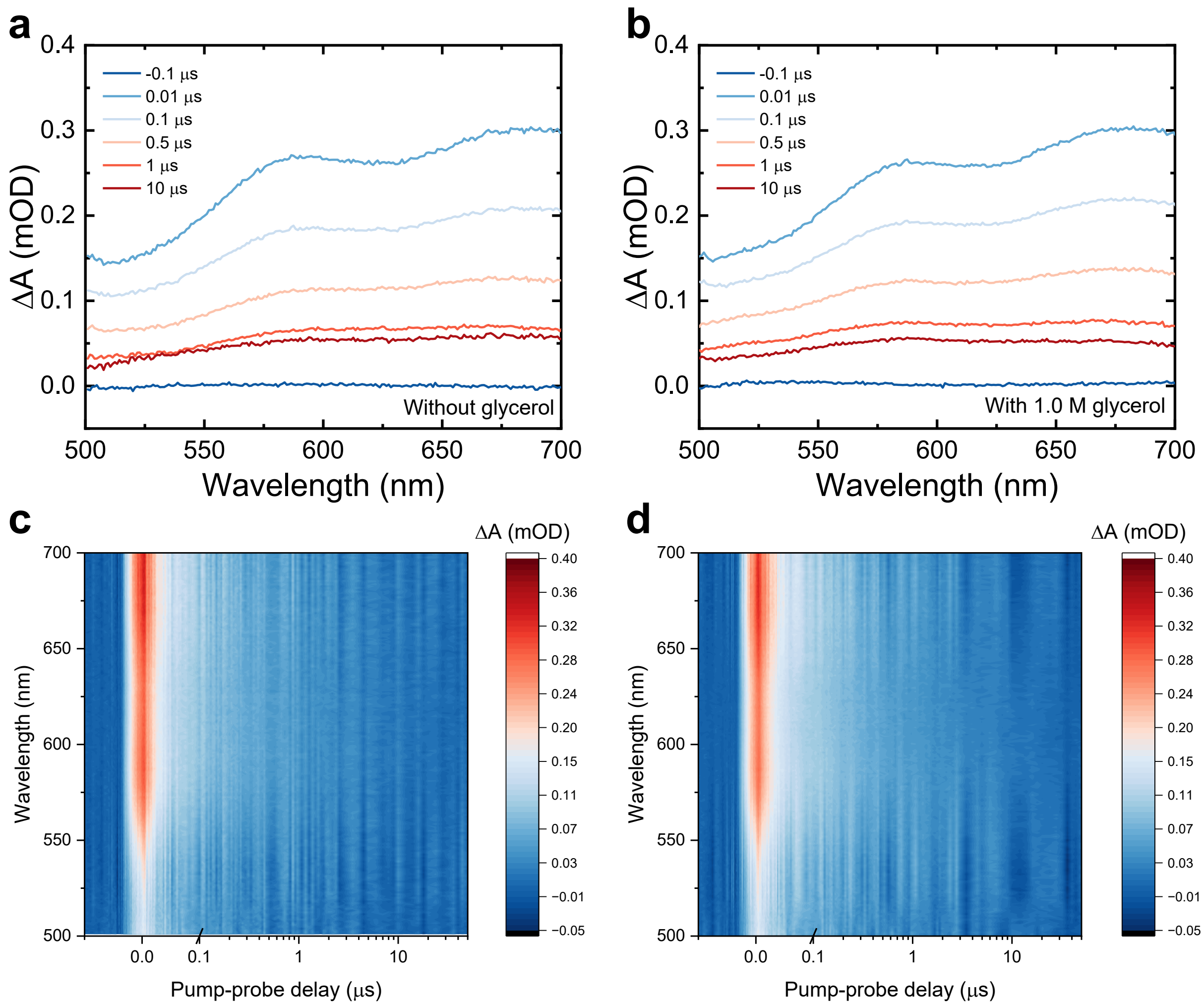

Figure S19. Representative ns-μs transient absorption (TA) spectra and spectral-temporal maps of $BiVO_4$ measured under the same operando electrolyte conditions as the 550 nm kinetics shown in **Fig. 4e**. (a,b) TA spectra at selected pump-probe delays from -0.1 μs to 10 μs measured in 0.5 M Na_2SO_4 (pH 6) (a) without glycerol and (b) with 1.0 M glycerol. (c,d) Corresponding spectral-temporal TA maps for $BiVO_4$ measured (c) without glycerol and (d) with 1.0 M glycerol.

References

(1) Tam, B.; Pike, S. D.; Nelson, J.; Kafizas, A. The scalable growth of high-performance nanostructured heterojunction photoanodes for applications in tandem photoelectrochemical-photovoltaic solar water splitting devices. *Chem. Sci.* **2025**, 16 (18), 7794–7810. DOI: 10.1039/D4SC08595G.

(2) Creasey, G. H.; McCallum, T. W.; Ai, G.; Tam, B.; Acosta, J. W. R.; Yousuf, A. M.; Fearn, S.; Eisner, F.; Kafizas, A.; Hankin, A. Mechanically and photoelectrochemically stable WO_3|$BiVO_4$| NiFeOOH photoanodes synthesised by a scalable chemical vapour deposition method. *J. Mater. Chem. A* **2025**, 13 (16), 11585–11604. DOI: 10.1039/D5TA00440C.

(3) Zhao, S.; Jia, C.; Shen, X.; Li, R.; Oldham, L.; Moss, B.; Tam, B.; Pike, S.; Harrison, N.; Ahmad, E. The aerosol-assisted chemical vapour deposition of Mo-doped $BiVO_4$ photoanodes for solar water splitting: an experimental and computational study. *J. Mater. Chem. A* **2024**, 12 (39), 26645–26666. DOI: 10.1039/D4TA02605E.

(4) Le Formal, F.; Pastor, E.; Tilley, S. D.; Mesa, C. A.; Pendlebury, S. R.; Grätzel, M.; Durrant, J. R. Rate law analysis of water oxidation on a hematite surface. *J. Am. Chem. Soc.* **2015**, 137 (20), 6629–6637. DOI: 10.1021/jacs.5b02576.

(5) Ma, Y.; Kafizas, A.; Pendlebury, S. R.; Le Formal, F.; Durrant, J. R. Photoinduced absorption spectroscopy of CoPi on $BiVO_4$: the function of CoPi during water oxidation. *Adv. Funct. Mater.* **2016**, 26 (27), 4951–4960. DOI: 10.1002/adfm.201600711.

(6) Ma, Y.; Pendlebury, S. R.; Reynal, A.; Le Formal, F.; Durrant, J. R. Dynamics of photogenerated holes in undoped $BiVO_4$ photoanodes for solar water oxidation. *Chem. Sci.* **2014**, 5 (8), 2964–2973. DOI: 10.1039/C4SC00469H.

(7) Ma, Y.; Mesa, C. A.; Pastor, E.; Kafizas, A.; Francàs, L.; Le Formal, F.; Pendlebury, S. R.; Durrant, J. R. Rate law analysis of water oxidation and hole scavenging on a $BiVO_4$ Photoanode. *ACS Energy Lett.* **2016**, *1* (3), 618–623. DOI: 10.1021/acsenergylett.6b00263.
